# Nanoscopic investigation of *C9orf72* poly-GA oligomers on nuclear membrane disruption by a photoinducible platform

**DOI:** 10.1038/s42004-021-00547-6

**Published:** 2021-07-23

**Authors:** Hung-Ming Chien, Ruei-Yu He, Chi-Chang Lee, Yung-An Huang, I-Ju Hung, Kai-Ting Hou, Jye-Chian Hsiao, Po-Chao Lu, Diksha Agnihotri, Eric Hwang, Joseph Jen-Tse Huang

**Affiliations:** 1 Institute of Chemistry, Academia Sinica, Taipei City, Taiwan; 2 Department of Chemistry, National Taiwan University, Taipei City, Taiwan; 3 Chemical Biology and Molecular Biophysics, Taiwan International Graduate Program, Academia Sinica and National Taiwan University, Taipei City, Taiwan; 4 Department of Biological Science and Technology, National Yang Ming Chiao Tung University, Hsinchu City, Taiwan; 5 Department of Chemical Engineering, National Taiwan University of Science and Technology, Taipei City, Taiwan; 6 Department and Graduate Institute of Pharmacology, National Taiwan University, Taipei City, Taiwan; 7 Taiwan International Graduate Program in Interdisciplinary Neuroscience, National Taiwan University and Academia Sinica, Taipei City, Taiwan; 8 Institute of Molecular Medicine and Bioengineering, National Yang Ming Chiao Tung University, Hsinchu City, Taiwan; 9 Institute of Bioinformatics and Systems Biology, National Yang Ming Chiao Tung University, Hsinchu City, Taiwan; 10 Center for Intelligent Drug Systems and Smart Bio-devices (IDS2B), National Yang Ming Chiao Tung University, Hsinchu City, Taiwan; 11 Department of Applied Chemistry, National Chiayi University, Chiayi City, Taiwan; 12 Neuroscience Program of Academia Sinica, Academia Sinica, Taipei, Taiwan

**Keywords:** Chemical tools, Nanostructures, Biophysical chemistry

## Abstract

Glycine-alanine dipeptide repeats (GA DPRs) translated from the mutated *C9orf72* gene have recently been correlated with amyotrophic lateral sclerosis (ALS). While GA DPRs aggregates have been suggested as amyloid, the biophysical features and cytotoxicity of GA DPRs oligomers has not been explored due to its unstable nature. In this study, we develop a photoinducible platform based on methoxynitrobenzene chemistry to enrich GA DPRs that allows monitoring the oligomerization process of GA DPRs in cells. By applying advanced microscopies, we examined the GA DPRs oligomerization process nanoscopically in a time-dependent manner. We provided direct evidences to demonstrate GA DPRs oligomers rather than nanofibrils disrupt nuclear membrane. Moreover, we found GA DPRs hamper nucleocytoplasmic transport in cells and cause cytosolic retention of TAR DNA-binding protein 43 in cortical neurons. Our results highlight the toxicity of GA DPRs oligomers, which is a key step toward elucidating the pathological roles of *C9orf72* DPRs.

## Abstract (web summary)

Glycine-alanine dipeptide repeats produced by the *C9orf72* gene are proposed to cause neurotoxicity in related diseases, but is challenging to study experimentally. Here a photolabile cell-penetrating peptide allows superresolution imaging of the distribution and aggregation of a simple dipeptide which recapitulates neurotoxicity associated with endogenous analogues.

## Introduction

G_4_C_2_ hexanucleotide repeat expansion in the noncoding region of the chromosome 9 open reading frame 72 (*C9orf72*) gene has been considered as the most prevalent genetic cause of amyotrophic lateral sclerosis (ALS) and frontotemporal dementia (FTD) recently^1–3^. Until now, three distinct mechanisms have been deduced^4–6^ to explain how G_4_C_2_ hexanucleotide expansion mutation confers neurotoxicity: (1) reduced *C9orf72* gene products owing to impaired transcription or splicing^7–9^; (2) G-quadruplex structure composed of the mutant RNA transcript to sequester essential RNA-binding proteins^10,11^; and (3) the unconventional dipeptide repeats (DPRs) translated from the sense and antisense strands of the RNA transcript^12–17^. Although these proposed pathomechanisms are not mutually exclusive, these DPRs, namely glycine–alanine (GA), glycine–arginine (GR), glycine–proline (GP), proline–alanine (PA), and proline–arginine (PR) have been, respectively, identified in ALS or FTD patients with *C9orf72* repeat expansions and thus drawn enormous attention^18,19^.

Among these DPRs, GA DPRs were the most abundant DPRs found in the *C9orf72*-mediated ALS patients^20,21^ and formed ubiquitin- and p62-positive inclusions in the cerebellum, hippocampus, and frontal cortex^22,23^. Although many DPRs have been proposed to participate in amyloid-like cascade in the disease progression^24^, only GA DPRs aggregates adopted beta-sheet conformation^25^. Revealing by another cryo-electron tomography study, GA DPRs inclusions are composed of amyloid-like ribbons^25^ and exhibited pervasive sequestration of proteasome^12,14,26^. Moreover, GA DPRs inclusions are also the perpetrator to blame for endoplasmic reticulum stress, synaptic-related protein sequestration^26,27^, and further neurotoxicity^21^. Collectively, this evidence suggested GA DPRs as a member of a growing list of misfolding agents in neurodegenerative diseases.

In the last two decades, the oligomeric species of misfolding proteins have emerged as a driver of neurotoxicity^28^. Despite its aggregation-prone propensity and ribbon-like conformation, the existence of GA DPRs oligomers during disease progression remains unknown. To recapitulate the accumulation of DPRs within cells, the ectopic gene overexpression was routinely practiced through AUG-mediated translation^12,14,18,29^. However, the bulky ectopic expression of GA DPRs may overshadow putative GA DPRs oligomers because the oligomeric intermediates would rapidly polymerize into mature fibrils. In addition, the instable expressivities of ectopically expressed products were also reported^17^ and extra tagging of reporters (e.g., GFP) may interfere with the intrinsic properties of GA DPRs^29^. Therefore, a controllable platform that provides a suitable window to observe GA DPRs oligomers in detail and characterize their proteinopathy in neurons is imperative.

**Fig. 1:**
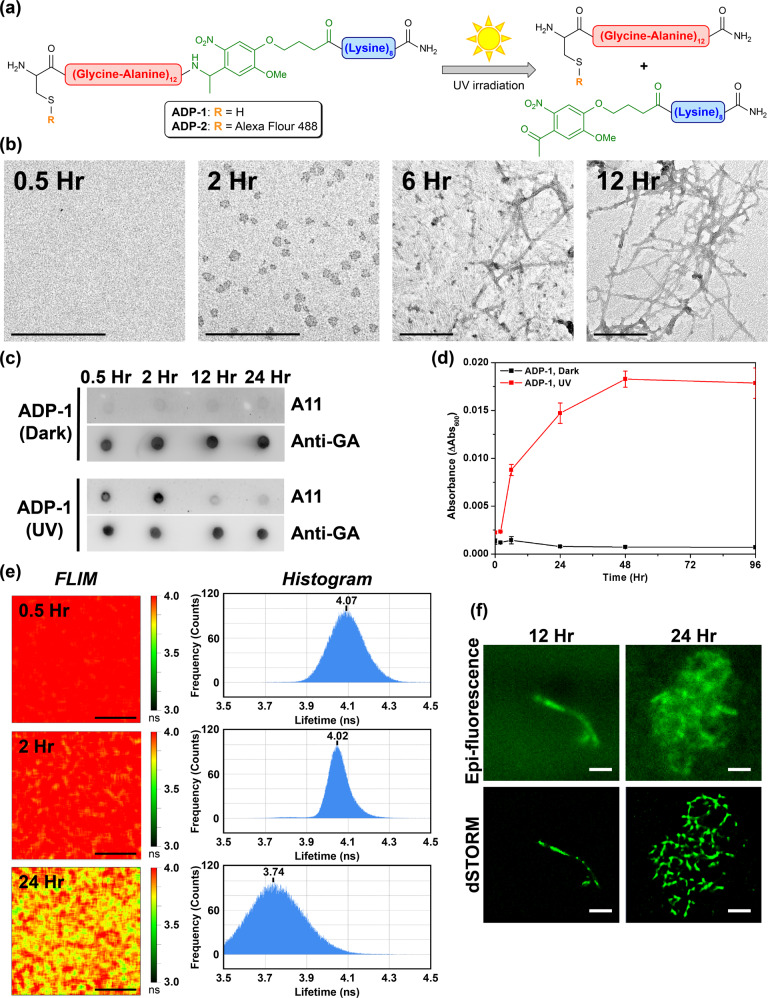
Diagram of ADP probes and their biophysical characterization. **a** Structure of ADP-1 and ADP-2 probes and the scheme of their photo-liberating reaction. **b** Time-course transmission electron microscope analysis of ADP-1 (50 μM). Scale bars indicate 200 nm. **c** Dot blot analysis of ADP-1-induced GA DPRs over time. The sample was incubated until the indicated time and applied on the membrane. Membranes were stained with anti-GA DPRs antibody and A11 antibody. *N* = 3. **d** Time-course turbidity measurements of irradiated (wavelength: 365 nm, power density: 32 mW/cm^2^, duration: 1 min) (red) and unirradiated (black) ADP-1 (50 μM). Data are collected at 0.5th, 2nd, 6th, 12th, 24th, 48th, and 96th hour (Standard deviation, *N* = 3). **e** Fluorescence lifetime images and the histograms of the photoinitiated ADP-2 (5 μM). Images were taken at the 0.5th, 2nd, and 24th hours of incubation; scale bars indicate 5 µm. **f** Epi-fluorescence and direct stochastic optical reconstruction microscopy (dSTORM) images of the photoinitiated ADP-2 (50 μM). Images were acquired at the 12th and 24th hours of incubation; scale bars indicate 10 µm.

To address the aforementioned questions, we have created photoinducible probes consisting of a cell-penetrating sequence (octalysine, K_8_), a GA DPRs [poly-(glycine–alanine), (GA)_12_] segment, a photolabile methoxynitrobenzene linker in the bridge (photolabile linker, PL), and a cysteine at N-terminus (ADP-1 in Fig. 1A). Through a Michael addition reaction with a photostable fluorophore (Alexa Fluor^TM^ 488 C5 Maleimide), we further prepared fluorescent probes for visualization (ADP-2 in Fig. 1a). This newly designed probe can easily penetrate cell membranes, prevent GA DPRs aggregation until photoinitiation, and induce GA DPRs oligomerization and fibrillization in neurons after photolysis. In comparison, we also synthesized probes with shorter repeats length GA DPRs [(GA)_3_], which could not oligomerize after photolysis as control (ADP-3 and ADP-4 in Figure S1). The repeat number of GA dipeptide in photoinducible probes was determined by the fact that (GA)_12_ but not (GA)_3_ formed amyloid nanofibrils (Supplementary Figs. 1–2). By exploiting this photoinducible platform, we characterized in-depth GA DPRs oligomerization and fibrillization process with both in vitro﻿ and ex vivo﻿ models in nanoscale. Furthermore, the impacts of GA DPRs on nuclear membrane integrity, nucleocytoplasmic transport, and neurite fragmentation have been explored.

## Results and discussion

### Photoinduced GA DPRs promptly form oligomers and subsequently turn into nanofibrils

Photoinducible ALS-associated dipeptides probes (ADP-1 to ADP-4) were synthesized and characterized as described in Methods Section (Fig. 1a, Supplementary Figs. 3–6, and Supplementary Table 1). To ensure ADP-1, [cysteine-(glycine–alanine)_12_-photolinker-lysine_8_], could undergo the photolysis reaction as shown in Fig. 1a, we irradiated ADP-1 (50 μM) solution with 365-nm light (power density: 32 mW/cm^2^; duration: 1 min) and then subjected to reversed-phase high-performance liquid chromatography (RP-HPLC) and mass spectroscopy analyses (Supplementary Fig. 3). Notably, two anticipated peptide fragments, respectively, octalysine (K_8_)-attached with photolinker (retention time ~14.5 min) and GA DPRs [(GA)_12_] (retention time ~23 min), were separated and identified (Supplementary Fig. 3).

After confirming the existence of the photoinduced GA DPRs, we next attempt to characterize their amyloid properties. Through the micrograph by transmission electron microscopy (TEM), we noticed liberated GA DPRs fragments promptly form spheroid aggregates in nanoscale at 2 h-incubation time and give rise to entangled nanofibrils (with the width ~10 nm) after 6 h while unirradiated ADP-1 form trace amorphous aggregates (Fig. 1b and Supplementary Fig. 7). Moreover, we have determined the hydrodynamic diameter of the spheroids aggregates with dynamic light scattering (DLS) and found the mean diameter (83 nm ± 19 nm) (Supplementary Fig. 8) is compatible with that observed in TEM (28 nm ± 8 nm). The hydrodynamic diameter measured by DLS better reflects the size of spheroids aggregates rather than the diameter found by TEM which is proceeding in dehydrated and near-vacuum environment^30^. On the basis of the amyloidogenic properties of GA DPRs, we surmised the spheroid aggregates at 2 h could be amyloid oligomers (Fig. 1b) and thus immunoblotted the ADP-1-derived GA DPRs with A11, an amyloid oligomers-specific antibody^31^, in a dot blot analysis. Interestingly, the A11-reactivity of the GA DPRs reached the strongest after 2 h incubation but soon receded as the incubation time extended, suggesting these transient spheroid aggregates are GA DPRs oligomers (Fig. 1c, Supplementary Fig. 9).

In addition to oligomerization, the rapid accumulation of the β-sheet content of the photoinitiated ADP-1 after 48 h incubation in circular dichroism (CD), as well as the enhanced fluorescence kinetic in thioflavin T (ThT)-binding assay over time, indicated the resultant GA DPRs underwent amyloidogenesis process (Supplementary Fig. 10a, b). However, though the minima at 220 nm can be commonly observed in amyloid filaments by CD, the β-sheet signature of these photoinitiated GA DPRs was not typical owing to the high abundance of achiral glycine in the sequence. To further characterize their secondary structure, we employed infrared spectroscopy and confirmed GA DPRs fibrils were enriched of β-sheet signature (Supplementary Fig. 10c, d)^32^. Moreover, in a time-course turbidity assay, the irradiated ADP-1 peaked within 48 h and remained high readouts through the observation window while the unirradiated ADP-1 remained low turbidity (Fig. 1d). In comparison, we have prepared another probe, ADP-3 [cysteine-(glycine–alanine)_3_-photolinker-lysine_8_] as a control. As observed by TEM, both the irradiated and unirradiated ADP-3 failed to grow nanofibrils and showed no significant turbidity increase even after 96 h incubation (Supplementary Fig. 11). Collectively, we demonstrated that GA DPRs fragments from ADP-1 probe are able to form amyloid oligomers and gradually turn into ThT-positive amyloid nanofibrils after photoinitiation.

### Visualizing GA DPRs oligomerization In vitro

Although we have detected GA DPRs oligomers and nanofibrils, respectively, in the photoinitiated ADP-1 as described above, we further exploited fluorescence lifetime imaging microscopy (FLIM) to monitor the aggregation process and elucidated their in-depth biophysical properties. As FLIM allows detailed monitoring of the differences in fluorescence lifetime fluorophores in response to the local environment and interactions, it has been applied to trace the aggregation kinetic process of fluorophore-attached proteins on the basis of fluorescence self-quenching property^33^. While the fluorescence lifetime dropping, it is manifesting that these proteins or peptides would form a highly compact structure. We thus decided to delineate the structural features of GA DPRs during oligomerization by applying time-course FLIM on the fluorophore-labeled probe, ADP-2 [Alexa Fluor 488-cysteine-(glycine–alanine)_12_-photolinker-lysine_8_]. As shown in Fig. 1e (upper panel), the average lifetime (*λ*) for monomeric AF-488-labeled GA DPRs was 4.07 ns after photolysis and 0.5 h incubation at 37°C. After 2 h incubation, ADP-2 exhibited an average lifetime of 4.02 ns with a number of puncta in FLIM, where denote the oligomeric species (Fig. 1e, middle panel). Moreover, after 24 h of incubation, as FLIM of ADP-2 showed a markedly patchy pattern and a much lower average lifetime (3.72 ns) along with a broadened distribution, hinting at the formation of nanofibrils (Fig. 1e, bottom panel). In addition, as a control, we measured the fluorescence lifetime of the fluorophore-labeled probe, ADP-4 [Alexa Fluor 488-cysteine-(glycine–alanine)_3_-photolinker-lysine_8_], in which the GA DPRs is much shorter. As expected, the fluorescence lifetime of irradiated ADP-4 remained at 4.1 ns through the whole time course (Supplementary Fig. 12). The decline of fluorescence lifetime in irradiated ADP-2 suggested that GA DPRs released from photoinitiated ADP-2 would form the higher-ordered aggregates, including oligomers and nanofibrils, in a time-dependent manner. Furthermore, we confirmed ADP-2-derived GA DPRs is capable of undergoing oligomerization and fibrillization process by TEM (Supplementary Fig. 13)

In addition, we applied direct stochastic optical reconstruction microscopy (dSTORM), which enables image acquisition at a resolution beyond the diffraction limit, to visualize GA DPRs fibrillization process at a submicron scale (Fig. 1f). After 12 h-incubation, GA DPRs developed linear aggregates with an average length of 20–50 μm. Moreover, clustered aggregates (ranging from 50 to 100 μm) were observed after 24 h-incubation. dSTORM revealed that aggregates were composed of fibrillar networks, which was comparable with foregoing TEM observation (Fig. 1b and Supplementary Fig. 13). Together with FLIM measurement, we made use of the fluorophore-labeled GA DPRs fragments from ADP-2 probe to visualize the oligomerization process and finally turned them into nanofibrils upon photoinitiation in a time course.

### ADP-1 can penetrate cells and induce GA DPRs oligomerization upon photoinitiation

**Fig. 2:**
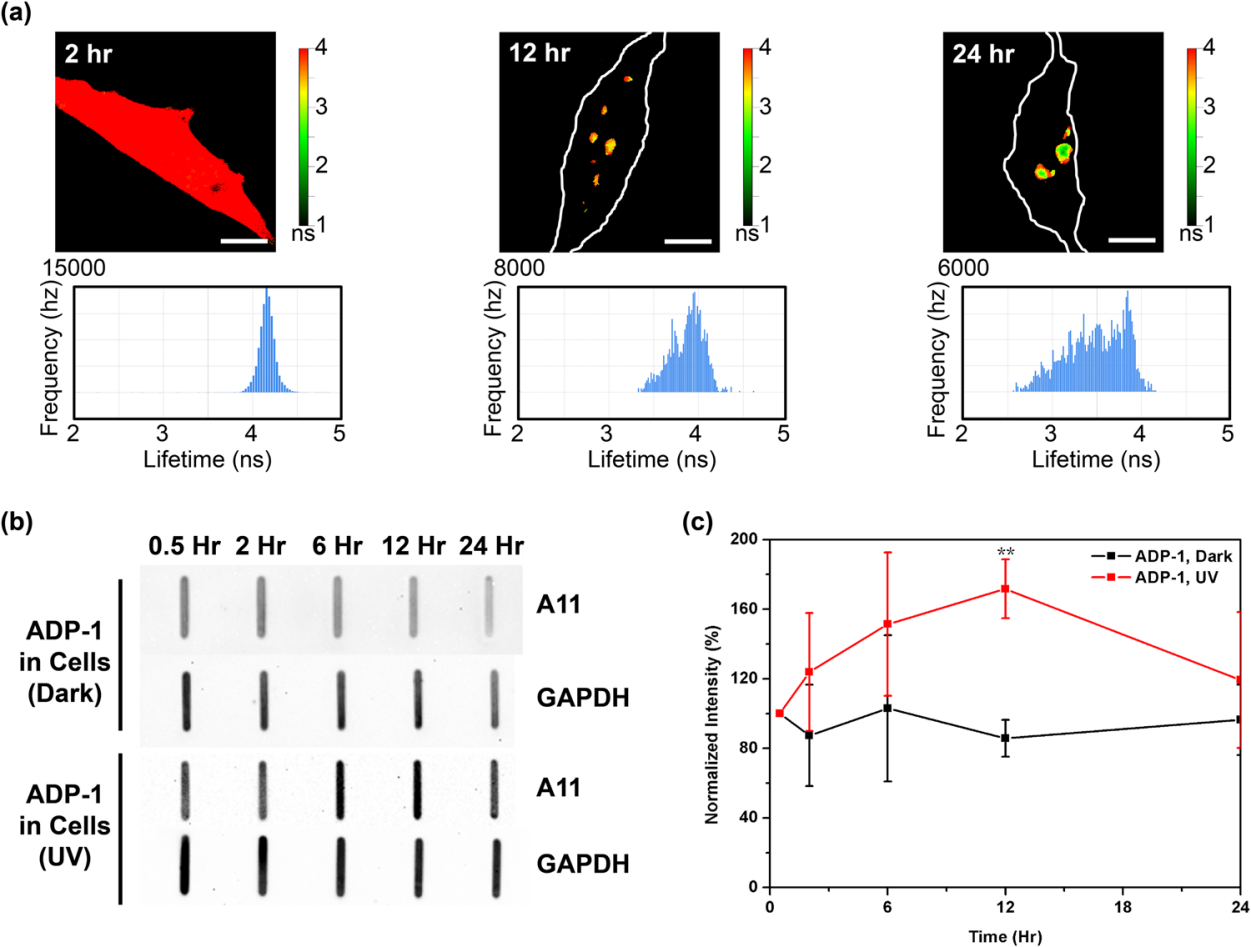
GA DPRs delivered by ADP-1 and 2 probes oligomerized in cells. **a** Time-course fluorescence lifetime images and histograms of ADP-2 in SH-SY5Y cells. 1 μM ADP-2 were treated to cells and photoinitiated by UV light (wavelength: 335–379 nm, power density: ≤8.24 mW/cm^2^, duration: 1 min). Cell periphery was contoured with a white line in 12th and 24th hours, respectively. Images were taken at 2nd, 12th, 24th hour after UV illumination; scale bars indicate 5 µm. **b** A11 immunoblotting with cell lysates from SH-SY5Y cells received ADP-1 (1 µM) treatment with or without photoinitiation. After photoinitiation, cell lysates at different time points were harvested and analyzed. **c** Quantification analysis of A11 immunoblot of SH-SY5Y total cell lysates. The signal of A11 staining was first normalized to GAPDH staining and then compared to signal at 0.5 h incubation. Three biological replicates were carried out (*r* = 3). Mean and standard deviation for ADP-1, UV = 171.7 ± 17.0; ADP-1, dark = 85.8 ± 10.6. ** indicates statistical significance where *p* value < 0.01 (analyzed by two-sided Welch’s *T* test, *p* value = 0.005, degree of freedom = 3, *t* value = 3.18).

As the biophysical properties of ADP-2 have been delineated in vitro, we next aimed to monitor the cell-penetration abilities as well as oligomerization and aggregation process of ADP-2 in neuronal-like cells (SH-SY5Y). As shown in FLIM, fluorescent GA DPRs released from ADP-2 were evenly distributed in cells after photoinitiation and 2 h incubation, with the corresponding average lifetime ~4.2 ns, suggesting GA DPRs remained in the monomeric states (Fig. 2a). As ADP-2-treated cell was incubated to 12 h, GA DPRs gradually assembled into submicron- and micron-sized puncta in the cytosol, with the lifetime decrease with uneven distribution (ranging from 3.5 to 4.2 ns; Fig. 2a). After 24 h incubation, GA DPRs developed highly compacted cytosolic inclusions, with the lifetime decreased further (2.5–4.1 ns; Fig. 2a). To further identify amyloid oligomers during GA DPRs oligomerization process in cells, we loaded the total lysates from ADP-1 treated SH-SY5Y cells with or without photoinitiation in a time-dependent manner on nitrocellulose membrane, and then immunoblotted with A11 antibody (Fig. 2b, details in SH-SY5Y cells lysates A11 staining in Methods section). The resultant A11 signal was first normalized to the internal control (GAPDH) and then compared with the signal at 0.5-hour-incubation (Fig. 2b). As the A11signals peaked from the 6th hour to 12th hours after photoinitiation, intracellular GA DPRs amyloid oligomers in ADP-1-treated cells are also transient, consistent with what we have observed in vitro (Fig. 2c). Instead, A11 signals in ADP-3-treated SH-SY5Y cell lysates remained mostly unchanged through the same incubation window (Supplementary Fig. 14), suggesting ADP-3 (with shorter GA DPRs) failed to induce GA DPRs oligomerization. It is noteworthy to mention that the slower GA DPRs oligomerization process in cells may result from the different concentrations used in experiments (1 μM for cellular experiment and 100 μM for in vitro immunostaining). In short, we have demonstrated photoinducible probes (ADP-1 and ADP-2) as a feasible platform to introduce cytosolic GA DPRs and initiate oligomerization in cells upon photoinitiation.

### Photoinitiated GA DPRs impaired nucleocytoplasmic transport in human neuroblastoma cells

**Fig. 3:**
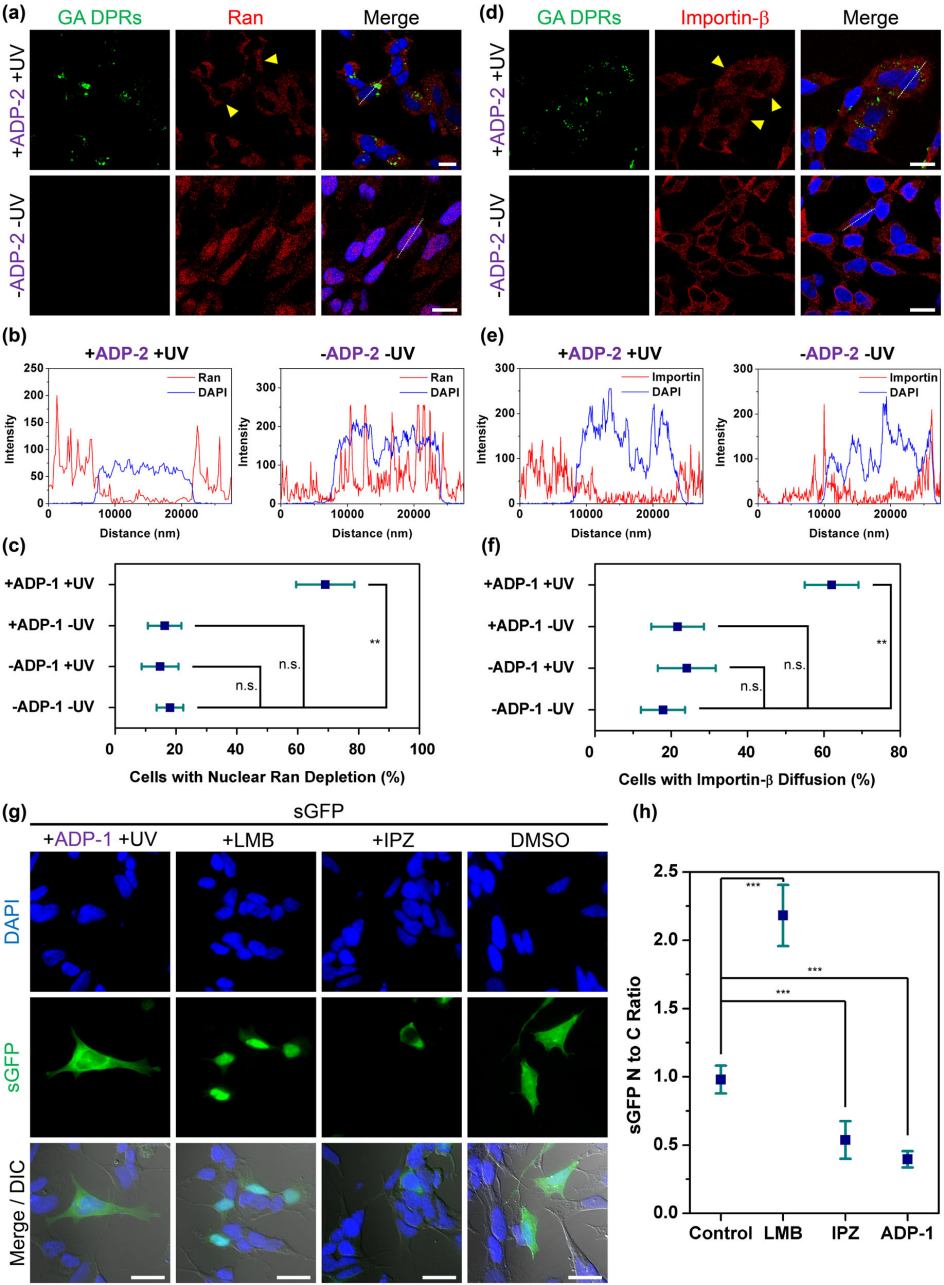
Photoinitiated GA DPRs impaired nucleocytoplasmic transport in human neuroblastoma cells. **a** Immunofluorescence images of Ran protein (red) in ADP-2 photoinitiated (+ADP-2 +UV) or control SH-SY5Y cells. GA DPRs aggregates were shown in green color. Cells were treated with ADP-2 (1 μM) and then exposed to UV light (wavelength: 335–379 nm, power density: ≤8.24 mW/cm^2^, duration: 1 min) and incubated for 24 h. Yellow arrows indicate the cells with nuclear Ran depletion. White dash line indicates the region for fluorescence intensity profiling. Scale bars indicate 10 µm. **b** Fluorescence intensity profile of selected cells in Fig. 3A. Blue curve indicates DAPI channel and red curve indicates Ran channel. **c** Quantification analysis of SH-SY5Y cells with nuclear ran protein depletion. Three biological replicates were carried out (*r* = 3). More than 55 cells were counted and analyzed in each group (*n* ≥ 55). Mean and standard deviation for +ADP-1 +UV = 69.0 ± 9.6; +ADP-1 −UV = 16.2 ± 5.5; −ADP-1 +UV = 14.7 ± 6.0; −ADP-1 −UV = 18.0 ± 4.4. ** indicates statistical significance where *p* value < 0.01 (analyzed by two-sided Welch’s *T* test with Bonferroni correction, test statistic in group comparison between +ADP-1 +UV and −ADP-1 −UV: *p* value = 0.0011, degree of freedom = 3, *t* value = 3.18; group comparisons between +ADP-1 −UV and −ADP-1 –UV: *p* value = 0.23, degree of freedom = 4, *t* value = 2.77; group comparison between −ADP-1 +UV and −ADP-1 –UV: *p* value = 0.16, degree of freedom = 4, *t* value = 2.77), and n.s. indicates not significant. **d** Immunofluorescence images of importin-β (red) in SH-SY5Y after ADP-2 (1 μM) and irradiation treatment. GA DPRs aggregates were shown in green color. Yellow arrows indicate the cells with importin-β mislocalization. White dash line indicates the region for fluorescence intensity profiling. Scale bars indicate 10 µm. **e** Fluorescence intensity profile of selected cells in Fig. 3D. Blue curve indicates DAPI channel and red curve indicate the importin-β channel. **f** Quantification analysis of SH-SY5Y cells with importin-β diffusion. Three biological replicates were carried out (*r* = 3). More than 37 cells were counted and analyzed in each group (*n* ≥ 37). Mean and standard deviation for +ADP-1 +UV = 62.0 ± 7.0; +ADP-1 −UV = 21.7 ± 6.9; −ADP-1 +UV = 24.1 ± 7.6; −ADP-1 −UV = 17.8 ± 5.8. *** indicates statistical significance where *p* value < 0.001 (analyzed by two-sided Welch’s *T* test with Bonferroni correction, test statistic in group comparison between +ADP-1 +UV and −ADP-1 −UV: *p* value = 0.0004, degree of freedom = 4, *t* value = 2.78; group comparison between +ADP-1 −UV and −ADP-1 –UV: *p* value = 0.17, degree of freedom = 4, *t* value = 2.77; group comparison between −ADP-1 +UV and −ADP-1 –UV: *p* value = 0.10, degree of freedom = 4, *t* value = 2.77), and n.s. indicates not significant. **g** Immunofluorescence images of sGFP-transfected SH-SY5Y cells after ADP-1 treatment (1 μM) and UV-irradiation. Corresponding nuclear importation (Importazole, IPZ, 40 µM) and exportation (leptomycin B, LMB, 20 nM) inhibitors were used here as reference groups, respectively. Scale bars indicate 10 µm. **h** Quantification analysis of the nuclear-to-cytoplasmic ratio of sGFP reporter in SH-SY5Y. One biological replicate were carried out (*r* = 1). Cell counting number (*n*) in each group: control = 38, LMB = 36, IPZ = 31, ADP-1 = 38. Mean and standard deviation for control = 0.98 ± 0.10; LMB = 2.18 ± 0.22; IPZ = 0.54 ± 0.14; ADP-1 = 0.40 ± 0.06. *** indicates statistical significance where *p* value < 0.001, (analyzed by two-sided Welch’s *T* test with Bonferroni correction, test statistic in group comparison between control and LMB: *p* value = 7.6 × 10^−33^, degree of freedom = 48, *t* value = 2.01; group comparison between control and IPZ: *p* value = 3 × 10^−21^, degree of freedom = 54, *t* value = 2.00; group comparison between control and ADP-1: *p* value = 2.6 × 10^−38^, degree of freedom = 59, *t* value = 2.00).

As the impairment of nucleocytoplasmic transport has a critical role in ALS^14,34,35^, we thus wonder if photoinitiated GA DPRs contribute similar phenotypes. We first examined the effect of GA DPRs on crucial cellular factors governing nuclear transport, including Ras-related nuclear protein (Ran) and importin-β. As an abundant protein in the nucleus, Ran drives nuclear transport via its concentration gradient between nucleus and cytoplasm^36^. After ADP-2 treatment followed by photoinitiation (hereafter referred to as ADP photoinitiated), we observed cytosolic retention of Ran protein accompanied by GA DPRs inclusions in SH-SY5Y cells (Fig. 3a), as demonstrated in the intensity profile (Fig. 3b). By contrast, Ran resided mainly in the nucleus in all control experiments (including the untreated, ADP-2-treated only, and photoinitiation only groups) (Fig. 3a–c, and Supplementary Fig. 15a), suggesting that the cytosolic GA DPRs may induce considerable Ran mislocalization into the cytoplasm. On the other hand, we also examined the effect of GA DPRs on importin-β, a client protein of Ran. Due to its weak binding affinity with FG-repeat domain within the nucleopore complex, importin-β is located mainly at perinuclear space^37^. We observed most importin-β escaped from the perinuclear region in the presence of photoinitiated GA DPRs. In comparison, all controls exhibited perinuclear importin-β (Fig. 3d–f, and Supplementary Fig. 15b). Moreover, we noticed that treating cells with the control peptide (ADP-3), which could not undergo oligomerization process, fail to initiate the mislocalization of the aforementioned transport factors (Supplementary Fig. 16). Taken together, our results showed ADP-2-derived GA DPRs interrupt nucleocytoplasmic transport regarding the mislocalization of Ran and importin-β.

In addition to mislocalization of transport factors, we thereafter suspected that whether if the bidirectional nucleocytoplasmic transport was impaired by GA DPRs. Consequently, we established a cargo distribution evaluation system by overexpressing green fluorescence proteins fused with both nuclear localization sequence (NLS) and nuclear export signal (NES) (denoted as shuttling GFP or sGFP) in SH-SY5Y cells (Fig. 3g) to investigate the nucleocytoplasmic transport function. As sGFP shuttle between nucleus and cytoplasm rapidly owing to its NLS and NES^15^, one could easily characterize the impairment of nuclear import and export by examining the subcellular distribution of sGFP in cells. To define the distribution of sGFP, we monitored the nuclear-to-cytoplasmic (N to C) ratio following the equation (N to C ratio = fluorescence intensity of sGFP in nucleus/fluorescence intensity of sGFP in the cytoplasm) through imaging analysis. It is expected the disruption of nuclear export will result in higher N to C ratio while the impairment of nuclear import will have a lower ratio than control (dimethyl sulfoxide). To validate this system, either nuclear import (Importazole, IPZ) or nuclear export inhibitor (leptomycin B, LMB) was applied as reference groups. As expected, whereas LMB caused most sGFP retained in the nucleus (N to C ratio = 2.2), IPZ led to sGFP accumulation in the cytosol (N to C ratio = 0.6; Fig. 3g, h). Notably, most of the sGFP remains in the cytosol upon ADP-1 treatment and photoinitiation, with the N to C ratio (0.5) being similar to that in IPZ (0.6; Fig. 3g, h), indicating the photoinitiated GA DPRs impaired nuclear import.

### GA DPRs oligomers can disrupt the nuclear membrane

**Fig. 4:**
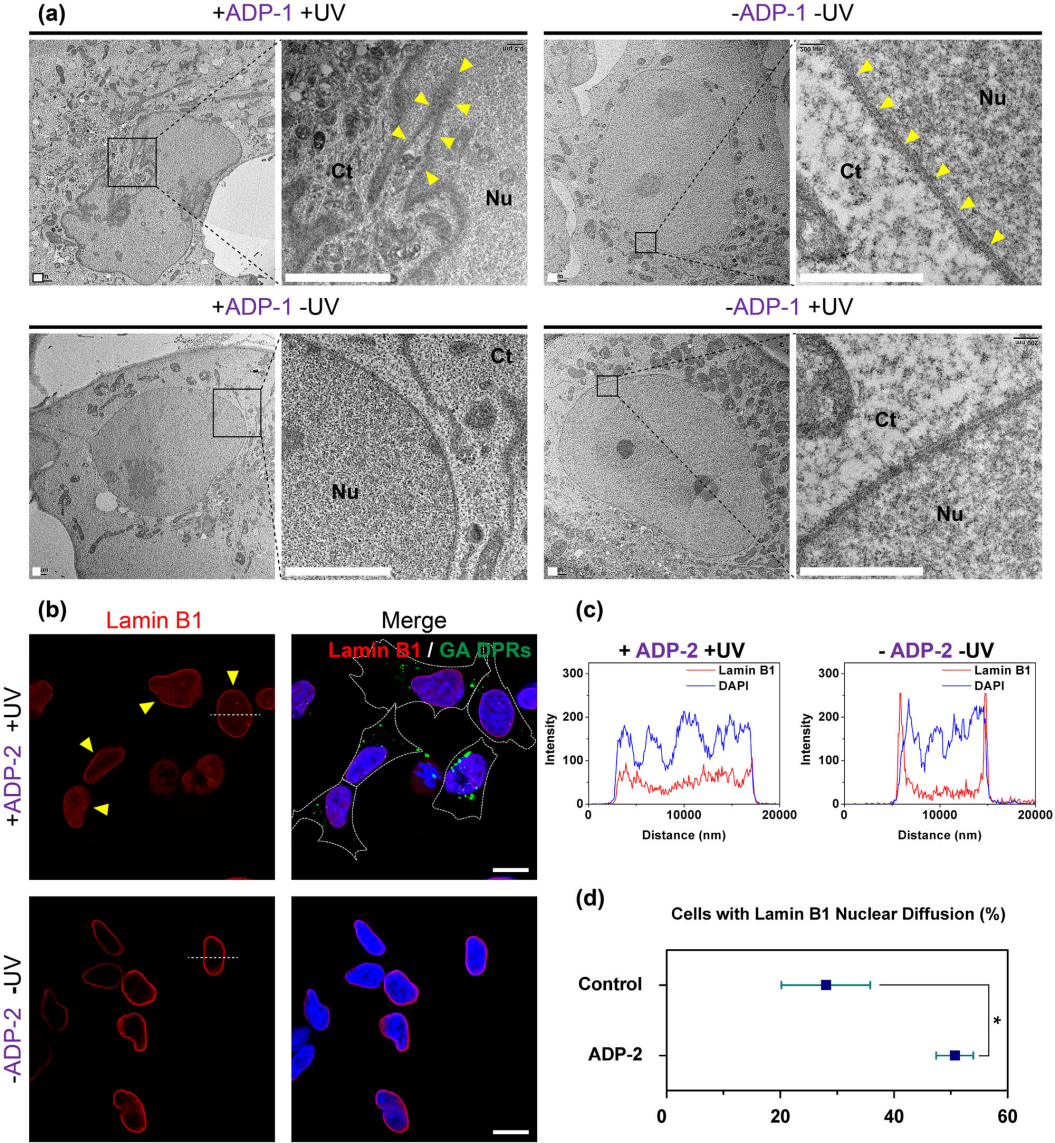
GA DPRs disrupt the nuclear membrane and lamina. **a** Transmission electron microscope images of in Cos-7 cells with ADP-1 treatment and photoinitiation, ADP-1 treatment only, photoinitiation only, and not treatment. Cos-7 cells were treated with ADP-1 (1 µM), photoinitiated, and then incubated for 24 h. Yellow arrows indicate the nuclear membrane. Black squares indicate the view of zoom-in images. Scale bars indicate 1 µm. **b** Immunofluorescence images of lamin B1 (red) in GA DPRs (green)-rich SH-SY5Y. Cells were treated with ADP-2 (1 µM), photoinitiated, and then incubated for 24 h. Yellow arrows indicate the lamin B1 nuclear diffusion. White dash line in the lamin B1 channel indicates the region for fluorescence intensity profiling. Scale bars indicate 10 µm. **c** Fluorescence intensity profile of the cells in Fig. 4B. Red curve indicates lamin B1 channel and the blue curve indicates DAPI channel. **d** Quantification analysis demonstrated the majority of cells with lamin B1 nuclear diffusion after ADP-2 treatment and photoinitiation (+ADP-2 +UV). Three biological replicates were carried out (*r* = 3). More than 53 cells were counted and analyzed in each group (*n* ≥ 53). Mean and standard deviation for group control = 28.0 ± 7.8; ADP-2 = 50.7 ± 3.3. * indicates statistical significance where *p* value < 0.05 (*p* value = 0.019, analyzed by two-sided Welch’s *T* test, degree of freedom = 3, *t* value = 3.18).

The nucleocytoplasmic transport dysregulation accompanied by nuclear membrane disruption has been linked with several neurodegenerative diseases^38–41^. Given that the expression of *C9orf72* mutations (G_4_C_2_ repeats) in cells resulted in membrane disruption^42^, we were intrigued whether GA DPRs can compromise membrane integrity. We thus examined the nuclear membrane ultrastructure in the presence and absence of ADP-1 under TEM. Cos-7 cell line was used here for better TEM imaging quality owing to its better attachment to the coverslip. The cells treated with ADP-1 displayed malformed nuclear morphology with folding and invaginations after photoinitiation (Fig. 4a). In particular, in higher magnification, the interspace of the nuclear membrane became blurred and uneven after ADP-1 treatment (+ADP-1 +UV), indicating GA DPRs compromise nuclear membrane integrity (indicated with yellow arrows). By contrast, the majority of control cells (including the untreated, ADP-1-treated only, and photoinitiation only groups) exhibited smooth and robust nuclear membrane structure, where the inner and outer nuclear membranes could be clearly identified in high magnification (Fig. 4a). Furthermore, we also examined whether nuclear lamina, a meshwork structure beneath the nuclear membrane and provides mechanical support^43^, would be affected in ADP-1-treated cells. By monitoring immunostaining of lamin B1, a nuclear lamina marker, we noticed the nuclear diffusion pattern of the lamina (indicated by yellow arrows) accompanied with GA DPRs inclusions (green) in ADP-2 photoinitiated cells (+ADP-2 +UV), which was seldom observed in controls (–ADP-2 –UV in Fig. 4b and +ADP-3 +UV in Supplementary Fig. 17). As shown in the intensity profile (Fig. 4c), lamin B1 is distributed through the nucleoplasm rather than tethering beneath the nuclear membrane. By quantifying the percentage of cells with lamin B1 nuclear diffusion staining pattern, we confirmed GA DPRs induced higher lamin B1 diffusion in nucleus pattern (51%) than control (23%) (Fig. 4d). Furthermore, through two-color dSTORM, we nanoscopically depicted inhomogeneous staining of lamin B1 along with the photoinitiated perinuclear GA DPRs inclusions (green) in the presence of ADP-2 (+ADP-2 +UV), whereas the smooth nuclear lamina (white) was observed in control (–ADP-2 –UV) (Supplementary Fig. 18). This morphological evidence indicated cells show severe defects in the nuclear periphery in response to GA DPRs.

**Fig. 5:**
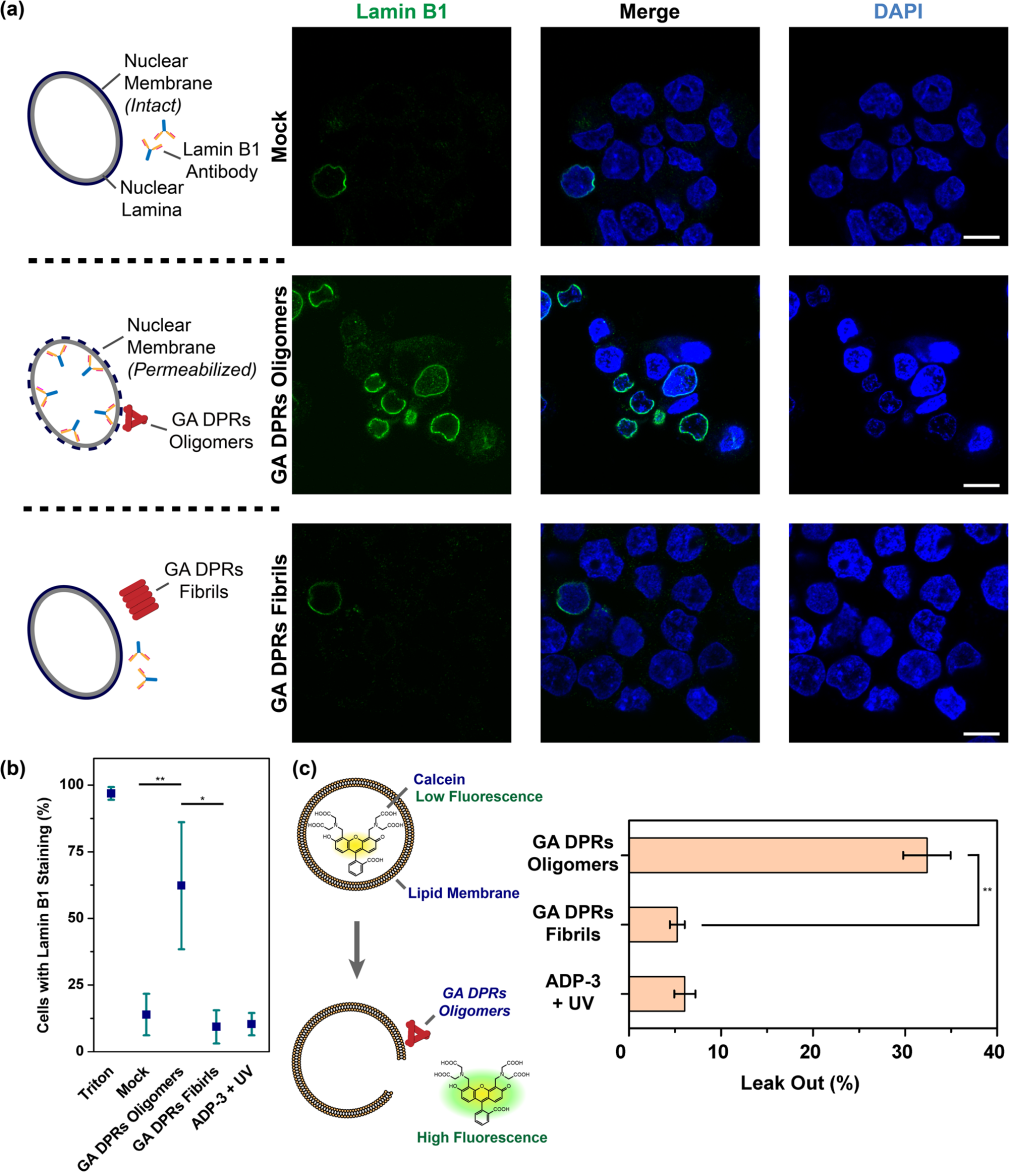
GA DPRs oligomers permeabilize the nuclear membrane. **a** Representative images of lamin B1 staining on digitonin-treated SH-SY5Y cells in the presence of GA DPRs oligomers (upper row), fibrils (middle), or mock (buffer only, bottom row). The nuclei were counterstained with DAPI. Scale bars indicate 10 μm. **b** Quantification analysis of SH-SY5Y cell nuclei with lamin B1 staining. Three biological replicates were carried out (*r* = 3). More than 27 cells were counted and analyzed in each group (*n* ≥ 27). Mean and standard deviation for mock = 13.9 ± 7.8; Triton = 96.9 ± 2.4; GA DPRs oligomers = 62.3 ± 23.9; GA DPRs fibrils = 9.5 ± 6.2; ADP-3 + UV = 10.6 ± 4.2. * indicates statistical significance where *p* value < 0.05 and ** indicates statistical significance where *p* value < 0.01 (analyzed by two-sided Welch’s *T* test with Bonferroni correction, test statistic in group comparison between mock and GA DPRs oligomers: *p* value = 0.009, degree of freedom = 4, *t* value = 2.77; group comparison between GA DPRs Oligomers and GA DPRs fibrils: *p* value = 0.011, degree of freedom = 3, *t* value = 3.18). **c** The ratio of the calcein (emission at 520 nm) leak-out from lipid-based liposome based on the fluorescence measurement. Fluorescence intensity was measured for each sample, and then subtracts from the background (liposome only) and normalized to the signal from the Triton X100 treatment (lysed liposome). Three experiment replicates were carried out (*r* = 3), Mean and standard deviation for GA DPRs oligomers = 32.4 ± 2.6; GA DPRs fibrils = 5.2 ± 0.8. ** indicates statistical significance where *p* value < 0.01 (analyzed by two-sided Welch’s *T* test, *p* value = 0.003, degree of freedom = 2, *t* value = 4.30).

Given the fact that the oligomers of misfolded protein readily impair membrane structure^44^, we conjectured that GA DPRs oligomers formed during the oligomerization process might be responsible for nuclear membrane disruption. To scrutinize the putative impingement of amyloid oligomers onto the nuclear membrane, we have pretreated SH-SY5Y cells with digitonin, a detergent that selectively permeabilizes plasma membrane but leaves the nuclear membrane intact^45^, to preserve the nucleus for the subsequent GA DPRs oligomers and nanofibrils treatments (details in ex vivo antibody penetrance assay in the Methods section). Since the intact nuclear membrane generally precludes the free passage of macromolecules like antibodies, the GA DPRs-treated nuclei were then immunostained with lamin B1 antibody, and the lamin B1 antibody penetrance was used to evaluate the corresponding membrane permeability (Supplementary Fig. 19). Different from the mock (upper row in Fig. 5a) and nanofibril-treated groups (bottom row in Fig. 5a), the oligomer-treated nuclei (middle row in Fig. 5a) exhibited the pronounced positive staining of lamin B1, revealing the GA DPRs oligomers enhance the penetrance of the nuclear membrane. Moreover, the ADP-3-treated group failed to induce significant lamin B1 staining, demonstrated that neither (GA)_3_ DPRs nor octalysine fragments were capable of impairing nuclear membrane (Supplementary Fig. 20). In order to evaluate the impact of GA DPRs on the nuclear membrane integrity, we defined: antibody penetrance level (%) = number of nuclei with lamin B1-positive staining/total nuclei numbers. We showed that the oligomers-treated group has a higher antibody penetrance level (62%) comparing with either the nanofibrils-treated group (9%), mock group (14%) or ADP-3-treated group (10.4%) (Fig. 5b). To further investigate the correlation between GA DPRs and nuclear membrane, we had also applied ADP-2-derived GA DPRs oligomers or fibrils in the aforementioned experiment. We noticed the fluorescent GA DPRs oligomers rather than fibrils attached to the nuclear membrane as confirmed by lamin B1 immunostaining (Supplementary Fig. 21). These findings suggested GA DPRs oligomers may directly interact with the nuclear membrane and compromise its integrity.

In order to clarify whether GA DPRs oligomers can disrupt lipid membranes, we monitored the fluorescence enhancement of calcein-encapsulated lipid-based liposomes in the presence and absence of different forms (either oligomers or nanofibrils) of GA DPRs. Since the fluorescence of calcein is self-quenched within the liposome but restored when calcein leaks out^46^, we herein define the membrane leak-out level (%) as the following. Leak-out (%) = [(enhanced fluorescence signal−background signal)/fluorescence signal with Triton X100] (details in Calcein-leakage assay in Methods section). We found GA DPRs oligomers photoinduced by ADP-1 cause the substantial release of liposomal content (~33%) within 2 h, whereas GA DPRs nanofibrils have a minimal effect (~6%) on calcein leakage (Fig. 5c). Notably, the control peptide, ADP-3, had a negligible effect (~7%) on the encapsulated liposome. In addition, we have also noticed that GA DPRs oligomers can directly interact with lipid-based liposomes (Supplementary Fig. 22). Collectively, we demonstrated the lipid membrane was disrupted mainly by GA DPRs oligomers rather than GA DPRs nanofibrils, short GA DPRs [(GA)_3_], or octalysine.

### GA DPRs induced endogenous TDP-43 cytosolic retention in human neuroblastoma

**Fig. 6:**
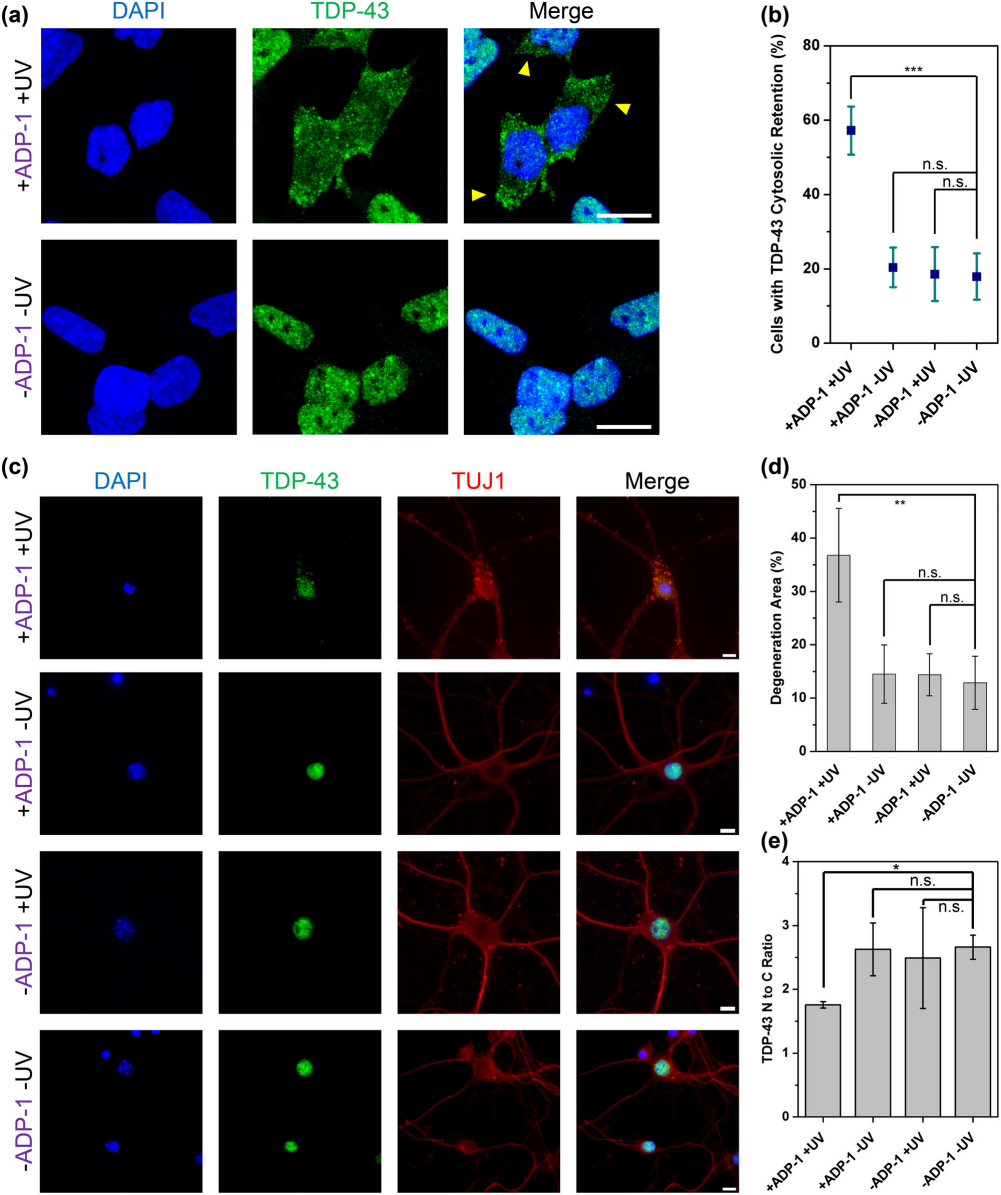
GA DPRs induces TDP-43 cytosolic retention and causes severe damage to the mouse cortical neurons. **a** Representive immunofluorescence of endogenous TDP-43 (Green) in neuroblastoma SH-SY5Y after ADP-1 treatment (1 μM), photoinitiated, and then incubated for 24 h. Yellow arrows indicate the cells with TDP-43 cytosolic retention. Scale bars indicate 10 µm. **b** Quantification analysis of SH-SY5Y cells with TDP-43 cytosolic retention. Three biological replicates were carried out (*r* = 3). More than 32 cells were counted and analyzed in each groups (*n* ≥ 32). Mean and standard deviation for +ADP-1 +UV = 57.2 ± 6.5; +ADP-1 −UV = 20.4 ± 5.4; −ADP-1 +UV = 18.6 ± 7.3; −ADP-1 −UV = 17.9 ± 6.3. *** indicates statistical significance where *p* value < 0.001 (analyzed by two-sided Welch’s *T* test with Bonferroni correction, test statistic in group comparison betwen +ADP-1 +UV and −ADP-1 −UV: *p* value = 0.0005, degree of freedom = 4, *t* value = 2.78; group compariosion betwen +ADP-1 −UV and −ADP-1 –UV: *p* value = 0.21, degree of freedom = 4, *t* value = 2.77; group compariosion betwen −ADP-1 +UV and −ADP-1 –UV: *p* value = 0.30, degree of freedom = 4, *t* value = 2.77), and n.s. indicates not significant. **c** Representative immunofluorescence images of 21DIV mouse cortical neurons stained with antibody against β-III-tubulin (red) and TDP-43 (green) treated with or without 1 μM ADP-1 and/or 2 min of UV irradiation. The mouse cortical neurons were further incubated for 24 h after ADP-1 treatment and/or UV irradiation. The DNA counter stain DAPI (blue) was included to identify the location of the nucleus. Scale bar indicates 10 µm. **d** Quantification analysis of degeneration area in 21DIV mouse cortical neurons. The degeneration area percentage (%) of neuron is quantified as fragemnted neurite area/total nerutite area. Three biological replicates were carried out (*r* = 3). Mean and standard deviation for +ADP-1 +UV = 36.8 ± 8.8; +ADP-1 −UV = 14.5 ± 5.5; −ADP-1 +UV = 14.4 ± 3.9; −ADP-1 −UV = 12.9 ± 5.0. ** indicates statistical significance where *p* value < 0.01 (analyzed by two-sided Welch’s *T* test with Bonferroni correction, test statistic in group compariosion betwen +ADP-1 +UV and −ADP-1 −UV: *p* value = 0.0086, degree of freedom = 3, *t* value = 3.18; group compariosion betwen +ADP-1 −UV and −ADP-1 –UV: *p* value = 0.24, degree of freedom = 4, *t* value = 2.77; group compariosion betwen −ADP-1 +UV and −ADP-1 –UV: *p* value = 0.234, degree of freedom = 4, *t* value = 2.77), and n.s. indicates not significant. **e** Quantification analysis nuclear-to-cytoplasmic ratio of TDP-43 in mice cortical neurons. Three biological replicates were carried out (*r* = 3) and counted neuron number (*n*) in each group: +ADP-1 +UV = 86; +ADP-1 −UV = 55; −ADP-1 +UV = 52; −ADP-1 −UV = 56. Mean and standard deviation for +ADP-1 +UV = 1.75 ± 0.05; +ADP-1 −UV = 2.62 ± 0.42; −ADP-1 +UV = 2.49 ± 0.79; −ADP-1 −UV = 2.66 ± 0.19. ** indicates statistical significance where *p* value  < 0.01 (analyzed by two-sided Welch’s *T* test with Bonferroni correction, test statistic in group compariosion betwen +ADP-1 +UV and −ADP-1 −UV: *p* value = 0.005, degree of freedom = 2, *t* value = 4.30; group compariosion betwen +ADP-1 −UV and −ADP-1 –UV: *p* value = 0.299, degree of freedom = 3, *t* value = 3.18; group compariosion betwen −ADP-1 +UV and −ADP-1 –UV: *p* value = 0.248, degree of freedom = 2, *t* value = 4.30), and n.s. indicates not significant.

In addition to DPRs, TAR DNA-binding protein 43 (TDP-43) proteinopathy is also considered as the hallmark in *C9orf72*-mediated ALS^47,48^. Studies have reported the cytosolic retention of TDP-43 induced by GA DPRs inclusions in HeLa cells^49,50^ and the co-accumulation of phosphorylated TDP-43 and GA DPRs proteins in neurons and diseased human brains^29,51^. On the basis of previous findings^52–54^, the association between GA DPRs inclusions and TDP-43 for *C9orf72*-mediated ALS pathogenesis is highly suspected, which drew our attention. In ADP-1-photoinitiated SH-SY5Y cells, through immunocytochemistry, we found most endogenous TDP-43 (green) translocated from the nucleus (blue) to the cytoplasm after photoinitiation (Top row in Fig. 6a). In contrast, TDP-43 resided in nuclei in all control groups (Fig. 6a, b, Supplementary Fig. 23). We thus reasoned endogenous TDP-43 cytosolic retention may result from the influence of GA DPRs-driven nuclear transport dysregulation.

### Introduction of GA DPRs caused severe damage to mouse cortical neurons

Studies have demonstrated that expressing GA DPRs in mouse cortical tissue induced cognitive deficits along with cerebellar atrophy, neural toxicity, and astrogliosis^14,55^. To examine the effect of GA DPRs on cortical neurons, ADP-1 was applied to dissociated primary cortical neurons which had been further cultured for 21 days in vitro. This prolonged culturing time allowed cortical neurons to mature and establish synaptic connections between each other^56^, effectively mimicking the physiological condition that occurs when *C9orf72*-mutation-mediated effects begin. Through immunofluorescence staining with neuron-specific β-III-tubulin antibody TUJ1 (details in ADP-1 treatment in mouse cortical neurons, immunofluorescence staining, and neurite fragmentation, and TDP-43 N to C ratio analysis in Supplementary Methods), we found the cortical neurons exhibited larger degeneration area percentage (fragmented TUJ1 area/total TUJ1 area) upon ADP-1 photoinitiation (Fig. 6c, d). In contrast to ADP-1-photoinitiated neurons, all control groups showed elaborate neurite arborization and minimal degeneration area percentage (including the untreated, ADP-1-treated only, and photoinitiation only groups) (Fig. 6c, d). We also observed the endogenous TDP-43 level decreased and became mislocalized from the nucleus to the cytoplasm. Through quantifying the N to C ratio of TDP-43 in the cortical neurons (Fig. 6e), we noticed the ADP-1 photoinitiated neurons exhibited a lower N to C ratio (1.75) compared with all the other control groups (2.5–2.7), indicating more TDP-43 translocated from the nucleus to the cytoplasm after photoinitiation. Taken together, we demonstrated photoinitiated ADP-1 caused the mislocalization of endogenous TDP-43 and the acute toxicity in primary cortical neurons, evincing GA DPRs induce cortical neuron degeneration.

## Conclusion

In summary, we successfully created a photoinducible platform to deliver GA DPRs into neurons. While amyloid oligomerization from monomers to nanofibrils is a quick equilibrium process, our platform underscores the possible roles of GA DPRs oligomers in *C9orf72*-mediated ALS. Through biophysical measurements, we comprehensively monitored the GA DPRs nanostructure and the oligomerization and fibrillization process in vitro and in neuronal-like cells. In respect of the observed nucleocytoplasmic transport dysregulation (e.g., Ran mislocalization and sGFP cytosolic accumulation), we reasoned that GA DPRs may induce nuclear membrane disruption. Along with ultrastructural studies, we confirmed GA DPRs perturbs the nuclear membrane by observing the invaginated nuclear membrane and nuclear diffusion of lamina upon ADP-1 photoinitiation. We probed the presence of amyloid oligomers in cells through the A11 immunoblotting. Given the amyloid oligomers tend to interfere with the lipid membrane, with further ex vivo and in vitro experiments, we have shown GA DPRs oligomers, rather than nanofibrils, disrupt the lipid membranes. In addition, aberrant cytosolic accumulation of endogenous TDP-43 in neurons through our probes showed the possible correlation of GA DPRs with TDP-43 in disease. Conclusively, we confirmed the nuclear membrane disruptive ability of photoinitiated GA DPRs oligomers and thus provided new insight into the possible pathological roles of GA DPRs, which may ultimately benefit the mechanistic studies of *C9orf72*-mediated ALS.

## Methods

### Polypeptide synthesis

Both amino acids and 4-[4-[1-(9-fluorenylmethyloxycarbonylamino)ethyl]-2-methoxy-5-nitrophenoxy]-n-butanoic acid (Fmoc-PL) were purchased from Advanced ChemTech. All peptides were synthesized by the standard Fmoc polyamide chemistry on Rink Amide AM resin (200–400 mesh, Merck-Millipore, Germany) using the automated peptide synthesizer Liberty Blue^TM^ (CEM, USA). Peptides were cleaved from the resin with cleavage cocktail (90% trifluoroacetic acid (TFA)/2.5% water/2.5% TIPS/5% EDT). Crude polypeptides were purified with HPLC (1260 Infinity LC system, Agilent, USA) equipped with a C18 reverse-phase semi-preparative column (Shiseido, Japan). The gradient separation was achieved by mixing buffer A (5% acetonitrile/0.1% TFA/94.9% water) and buffer B (0.1% TFA/99.9% acetonitrile). Polypeptide purity was confirmed by RP-HPLC with an analytical column (C18) and identified by matrix-assisted laser desorption/ionization (Applied Biosystem, USA) mass spectroscopy.

### Fluorescence-lifetime imaging microscopy

Based on the self-quenching of the neighboring fluorophores labeled on the GA DPRs peptide photo released from ADP-2, FLIM analysis was carried out with nanometer resolution Z-piezo objective. The laser for excitation was 488 nm modulated laser line with 20 MHz repetition rate. The fluorescence lifetime of Alexa Fluor 488-labelled GA DPRs peptides with different incubation times were monitored and compared. The excitation wavelength was connected by optical fiber and a bandpass filter to improve wavelength selection. Fluorescence emission from the sample went through a bandpass filter (FF05-500/25-25 for Alexa 488, Semrock) before being sent to the confocal unit with single-photon counting module avalanche photodiodes APDs detectors. A water objective (PlanApo 60×, N.A. 1.2, Nikon) was used for the imaging. To avoid any perturbation to the sample, optical sectioning was achieved by the XY galvo mirror scanning and the Z sectioning by moving Piezo encoded motorized device. For the cellular FLIM images, SH-SY5Y cells were cultured on a sterilized 35 mm glass-bottom μ-Dish (Ibidi). After ADP-2 treatment and photoinitiation (see below), cells were further incubated for 2, 12, and 24 h followed by 4% paraformaldehyde fixation. FLIM analysis was carried out essentially as described above.

### SH-SY5Y cells lysates A11 staining

In all, 1.6 × 10^6^ of SH-SY5Y cells were treated with ADP-1 or ADP-3 (3 μM) for 24 h and then exposed to UV light (mercury lamp with 345−385 nm bandpass filter; average power: 8.24 mW/cm^2^; duration: 3 min). After washing with culture medium (Dulbecco’s Modified Eagle Medium/F12 supplemented with 10% FBS), cells were further incubated and collected the cell lysates in RIPA buffer at different time points. Cells lysates were first sonicated by UP200S (Hielscher Ultrasonics, Germany) and the total protein concentration in the resultant lysates was measured by detergent compatible protein assay (Bio-Rad, USA). In all, 50 μg of proteins were loaded onto nitrocellulose membrane (0.1 μm, GE healthcare, USA) adopted with the PR648 Slot Blot Blotting Manifold. Collected membranes were blocked by 2% bovine serum albumin and then stained with A11 antibody (AHB0052, ThermoFisher) and GAPDH antibody (GTX627408, GeneTex), respectively. To quantitate the A11 kinetics, the collected A11 signals were first normalized to internal control GAPDH and the resultant signals were then compared to the signal at 0.5 h-incubation to learn the relative fold value.

### Ex vivo antibody penetrance assay

To prepare the GA DPRs oligomers-rich samples, ADP-1 (100 μM in phosphate-buffered saline; PBS) solution was irradiated by UV light (365 nm, 1200 μw/cm^2^, duration: 1 min) and incubated for 2 h at 37°C. For the GA DPRs fibrils-rich samples, ADP-1 (100 μM in PBS) solution was first irradiated by UV light and incubated for 24 h at 37°C. The resulting GA DPRs fibrils were collected by centrifugation (16,000 × *g*) for 30 min. Collected pellets were resuspended in PBS buffer and sonicated for 1 h by sonicator (D150H, DELTA Ultrasonic Cleaner, Taiwan) before treating nuclear remains.

For plasma membrane permeabilization, 2 × 10^6^ of SH-SY5Y cells were pretreated with icy digitonin (0.001% in PBS) for 2 min. The resulting nuclei were washed by icy PBS three times and then treated with GA DPRs oligomers or fibrils for 2 h at 37°C. After PBS rinses, the permeabilized remains were fixed and stained with lamin B1 antibody. Confocal images of the resulting samples were captured with LSM 780. The antibody penetrance level in the nuclei after digitonin treatment was evaluated by the number of nuclei with positive staining of lamin B1 over total nuclei number.

### Calcein-leakage assay

For preparation of calcein-containing liposome, 1,2-Ditetradecanoyl-sn-glycero-3-phospho-(1’-rac-glycerol) (sodium salt) (3.3 mg), 1,2-Dimyristoyl-sn-glycero-3-phosphocholine (5.0 mg), and cholesterol (2.5 mg) were dissolved in 1:1 chloroform/methanol. Nitrogen gas and lyophilizer were used to remove the solvents from the sample. The dried lipid was rehydrated by 1 ml of 50 mM calcein-containing PBS titrated with potassium hydroxide to pH 7.0. The solution was sonicated for 1 h. Freeze-thaw cycles in liquid nitrogen and a 70°C hot plate were processed seven times. The suspension was passed back and forth continuously through an Avanti Mini-Extruder with two stacked 100 nm polycarbonate membranes (Avanti Polar Lipid, Inc). Unencapsulated calcein was removed by a self-packed size-exclusion column with Sepharose CL-4B gel filtration medium (Sigma). For measuring the fluorescence intensity, peptides (100 μM) were freshly prepared and then added into the liposome solution to a final concentration of 0.7 mM lipid and 50 μM peptide. For collecting GA DPRs fibrils, the irradiated ADP-1 was incubated for 1 day, centrifuged. The pellet was re-suspended before adding to liposome solution. The liposome and peptide mixtures were incubated at 37°C with 1400 rpm shaking in an Eppendorf Thermomixer® mixer/incubator. Fluorescence was measured at excitation/emission 490/520 nm. The percentage of fluorescence intensity is defined as (FP−FL)/(FT−FL), where FP is the fluorescence signal after the addition of peptides, FL is the fluorescence for liposome only, and FT is the fluorescence signal obtained after the addition of 5% of Triton X100.

### Reporting summary

Further information on research design is available in the Nature Research Reporting Summary linked to this article.

## Supplementary information

Description of Additional Supplementary Files

Supplementary Information

Supplementary Data 1

Reporting Summary

## Data Availability

The authors declare that all data supporting the findings of this study are available within the paper and its supplementary files (Supplementary Information). The raw statistical data, two-sided Welch’s T test statistics, and multiple comparison correction results are included in Supplementary Data 1.
