# Supplementary material for: Nanoscopic investigation of C9orf72 poly-GA oligomers on nuclear membrane disruption by a photoinducible platform
Source: Commun Chem. 2021 Jul 23;4:111. doi: 10.1038/s42004-021-00547-6 (PMC9814621; doi:10.1038/s42004-021-00547-6)
Supplement: Supplementary file 1 — Description of Additional Supplementary Files [file 42004_2021_547_MOESM1_ESM.pdf]

Description of Additional Supplementary Files

File Name: Supplementary Data 1

Description: This Microsoft Excel file includes raw statistical data from experiments, two-sided Welch's T test's statistics, and multiple comparison correction results.
