# Supplementary material for: Nanoscopic investigation of C9orf72 poly-GA oligomers on nuclear membrane disruption by a photoinducible platform
Source: Commun Chem. 2021 Jul 23;4:111. doi: 10.1038/s42004-021-00547-6 (PMC9814621; doi:10.1038/s42004-021-00547-6)
Supplement: Supplementary file 2 — Supplementary Information [file 42004_2021_547_MOESM2_ESM.pdf]

Supplementary Information

Nanoscopic investigation of *C9orf72* poly-GA oligomers on nuclear membrane disruption by a photoinducible platform

Authorship

*Hung-Ming Chien[1, 2, 3], Ruei-Yu He[1], Chi-Chang Lee[1], Yung-An Huang[1, 4], I-Ju Hung[1, 5], Kai-Ting Hou[1], Jye-Chian Hsiao[1], Po-Chao Lu[1, 3, 6], Diksha Agnihotri[1, 7], Eric Hwang[4, 8, 9, 10], and Joseph Jen-Tse Huang[1, 3, 11, 12 *]*

Affiliation:

[1]Institute of Chemistry, Academia Sinica, No. 128, Sec. 2, Academia Rd., Nangang Dist., Taipei City, Taiwan

[2]Department of Chemistry, National Taiwan University, No. 1, Sec. 4, Roosevelt Rd., Da'an Dist., Taipei City, Taiwan

[3]Chemical Biology and Molecular Biophysics, Taiwan International Graduate Program, Academia Sinica and National Taiwan University, No. 128, Sec. 2, Academia Rd., Nangang Dist., Taipei City, Taiwan

[4]Department of Biological Science and Technology, National Yang Ming Chiao Tung University, No. 1001, University Rd., Hsinchu City, Taiwan.

[5]Department of Chemical Engineering, National Taiwan University of Science and Technology, No. 43, Sec. 4, Keelung Rd., Da'an Dist., Taipei City, Taiwan

[6]Department and Graduate Institute of Pharmacology, National Taiwan University, No. 1, Sec. 4, Roosevelt Rd., Da'an Dist., Taipei City, Taiwan

[7]Taiwan International Graduate Program in Interdisciplinary Neuroscience, National Taiwan University and Academia Sinica, No. 128, Sec. 2, Academia Rd., Nangang Dist., Taipei City, Taiwan

[8]Institute of Molecular Medicine and Bioengineering, National Yang Ming Chiao Tung University, No. 1001, University Rd., Hsinchu City, Taiwan

[9]Institute of Bioinformatics and Systems Biology, National Yang Ming Chiao Tung University, No. 1001, University Rd., Hsinchu City, Taiwan

[10]Center for Intelligent Drug Systems and Smart Bio-devices (IDS2B), National Yang Ming Chiao Tung University, No. 1001, University Rd., Hsinchu City, Taiwan

[11]Department of Applied Chemistry, National Chiayi University, No. 300, Xuefu Rd., East Dist., Chiayi City, Taiwan

[12]Neuroscience Program of Academia Sinica, Academia Sinica, No. 128, Sec. 2, Academia Rd., Nangang Dist., Taipei, Taiwan

Corresponding author email address:

jthuang@gate.sinica.edu.tw

Supplementary Figures

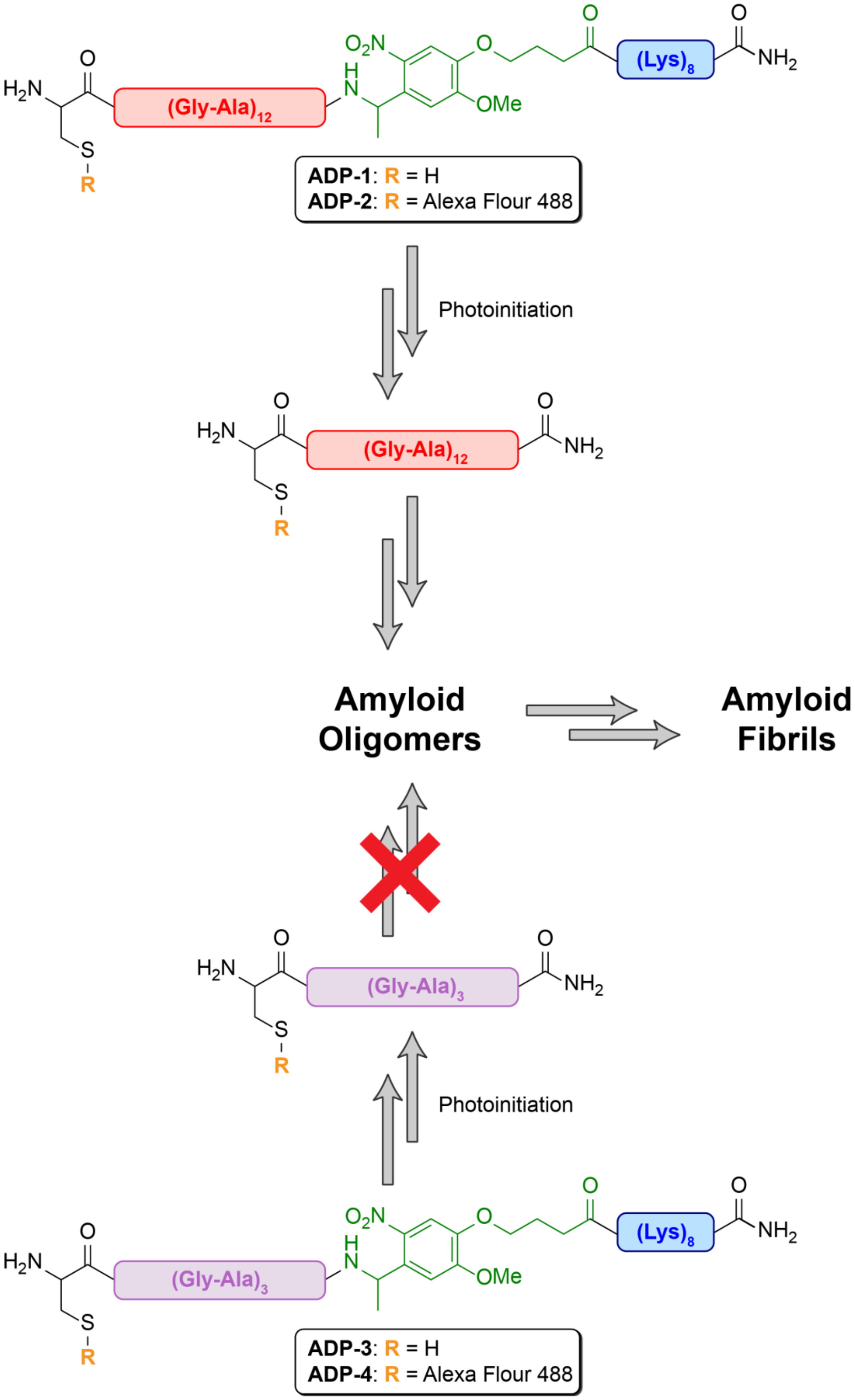

Supplementary Figure 1. Schematic figure for ADP probes.

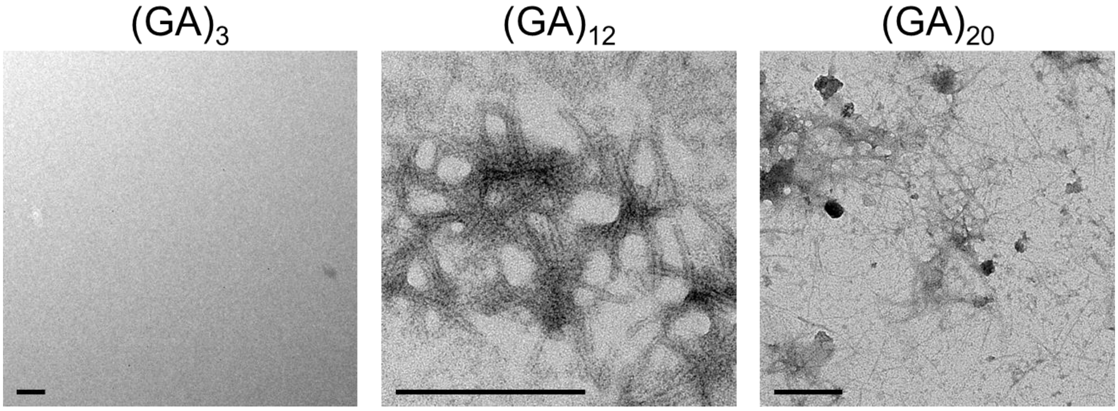

Supplementary Figure 2. TEM images of GA DPRs with varied chain length. Peptides (50 μM) were prepared in the low salt phosphate buffer saline (0.05 M NaCl, 0.0027 M KCl, 0.01 M Na_2HPO_4, 0.002 M KH_2PO_4, pH = 7.4) and incubated at 37 °C for one day. Scale bar indicates 200 nm.

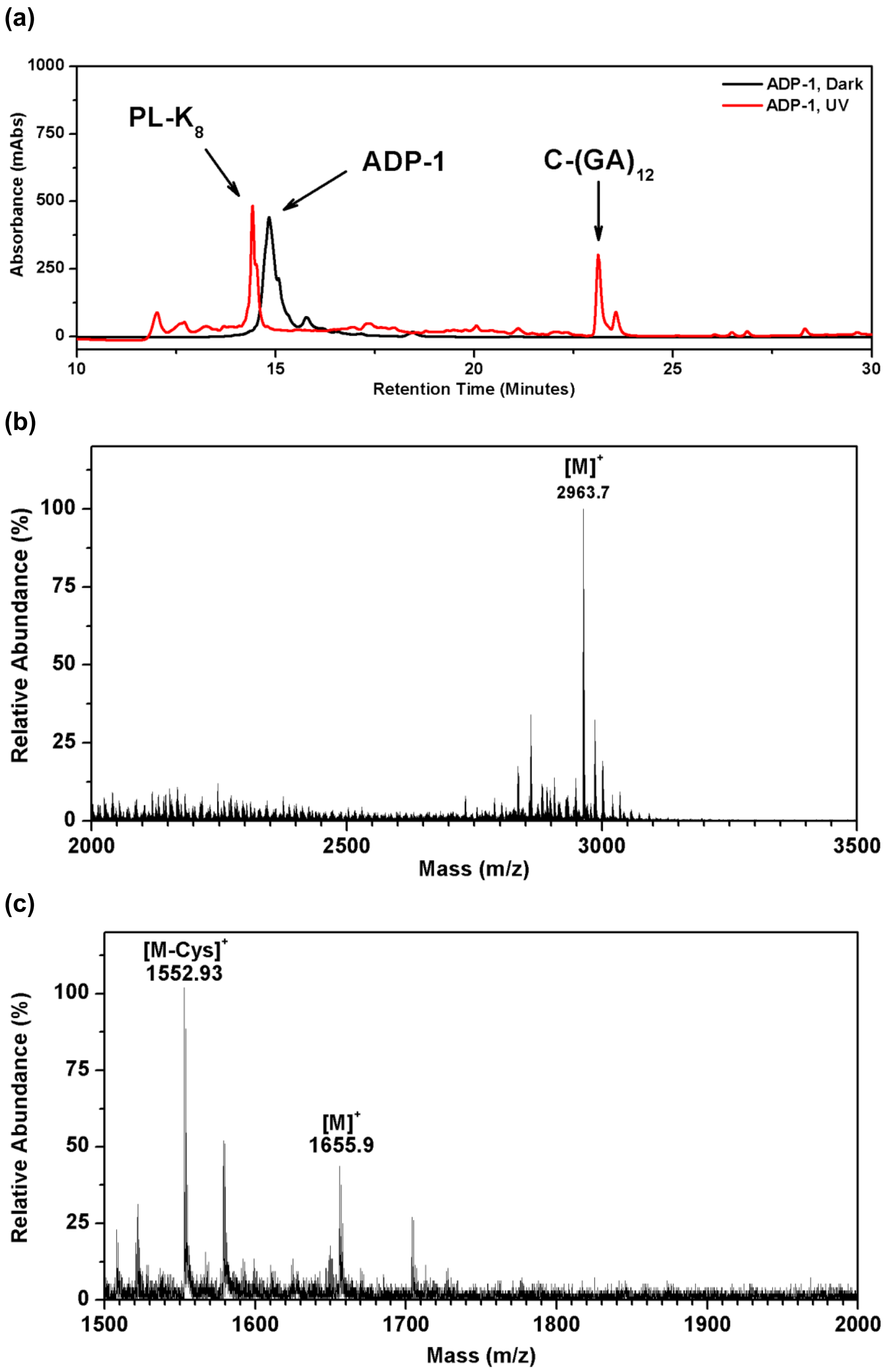
(a)
ADP-1, Dark
ADP-1, UV
PL-K8
ADP-1
C-(GA)12
Absorbance (mAbs)
Retention Time (Minutes)
(b)
[M]+
2963.7
Relative Abundance (%)
Mass (m/z)
(c)
[M-Cys]+
1552.93
[M]+
1655.9
Relative Abundance (%)
Mass (m/z)

(d)

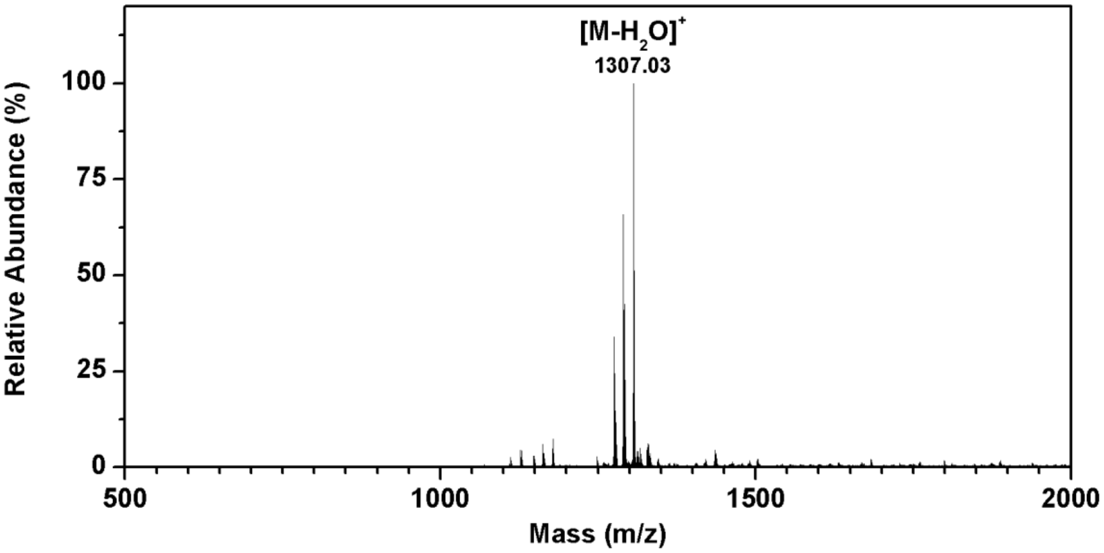

Supplementary Figure 3. HPLC and MS spectra of ADP-1. (a) High performance liquid chromatography spectra of unirradiated and irradiated ADP-1. The first red peak with retention time around 14 minute is the photolinker-octalysine segment as we confirmed by mass in Supplementary Figure 3d, whereas the other red peak with retention time about 23 minute is the GA DPRs fragment as we identified by mass in Supplementary Figure 3c. **(b)** Matrix-assisted laser desorption/ionization-time of flight mass spectra of ADP-1, calc. mass: 2963.7; observed: 2963.7 ($[M]^+$). **(c)** Matrix-assisted laser desorption/ionization-time of flight mass spectra of GA DPRs fragment from the photoinitiated ADP-1, calc. mass: 1657; observed: 1655.9 ($[M]^+$). **(d)** Matrix-assisted laser desorption/ionization-time of flight mass spectra of octalysine fragment from the photoinitiated ADP-1, calc. mass: 1324.3 ; observed:1307.03 ($[M-H_2O]^+$).

(a)

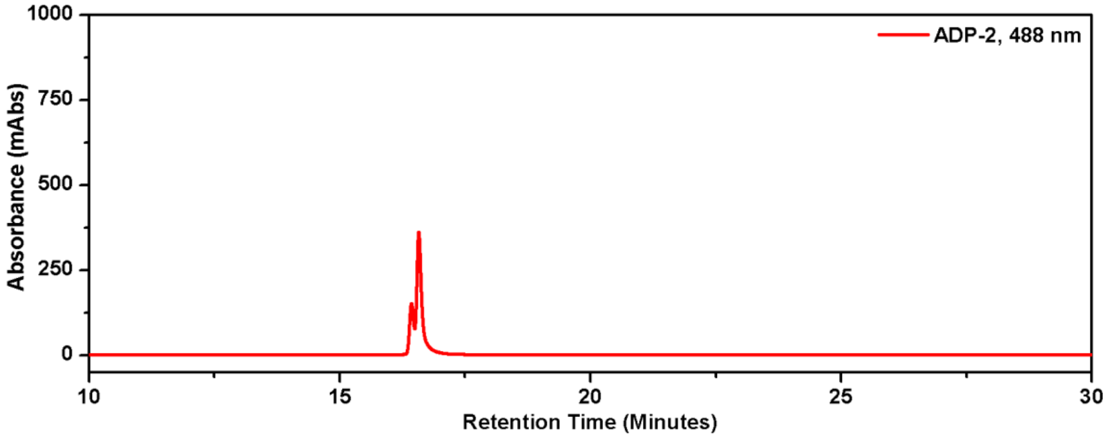

(b)

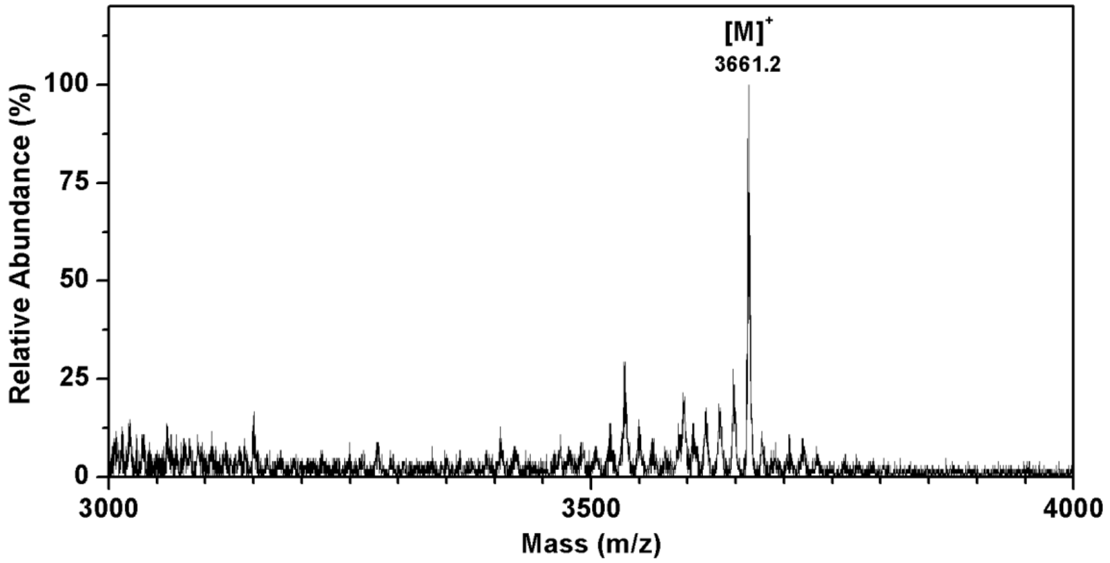

Supplementary Figure 4. HPLC and MS spectra of ADP-2. (a) High performance liquid chromatography spectra of ADP-2 **(b)** Matrix-assisted laser desorption/ionization-time of flight mass spectra of ADP-2, calc. mass: 3661.0; observed: 3661.2. ($[M]^+$).

(a)

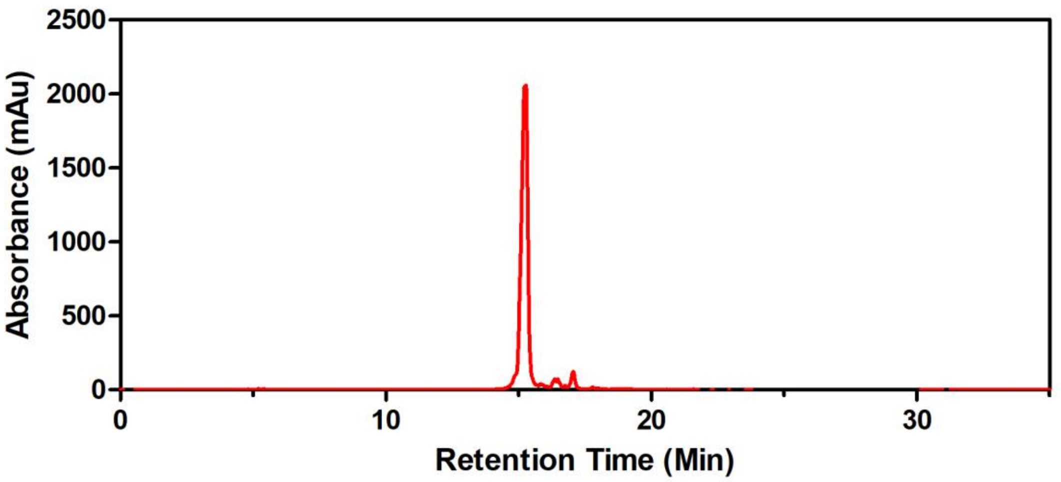

(b)

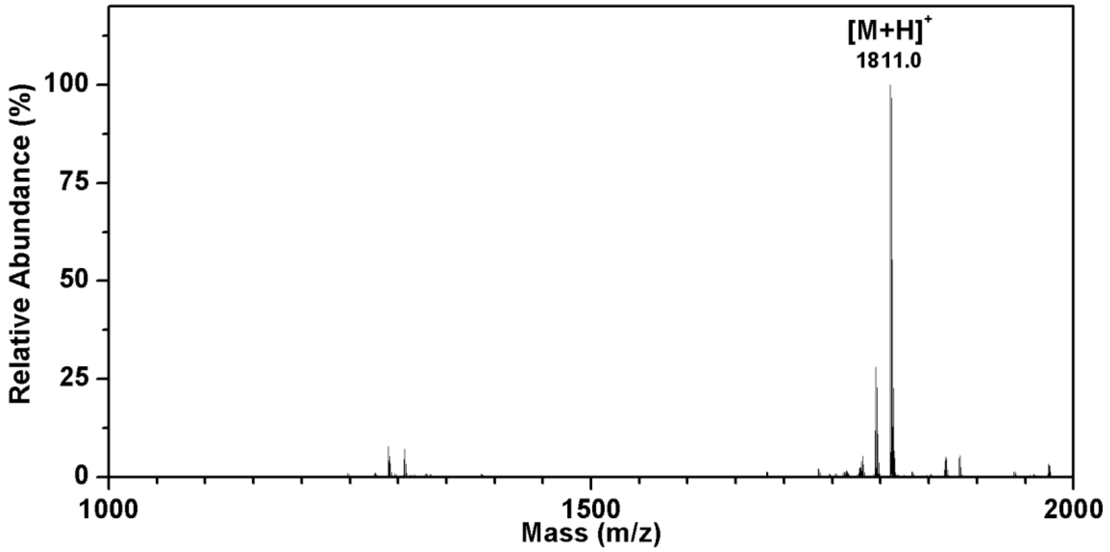

Supplementary Figure 5. HPLC and MS spectra of ADP-3. (a) High performance liquid chromatography spectra of ADP-3 **(b)** Matrix-assisted laser desorption/ionization-time of flight mass spectra of ADP-3, calc. mass: 1809.9; observed: 1811.0 ($[M+H]^+$).

(a)

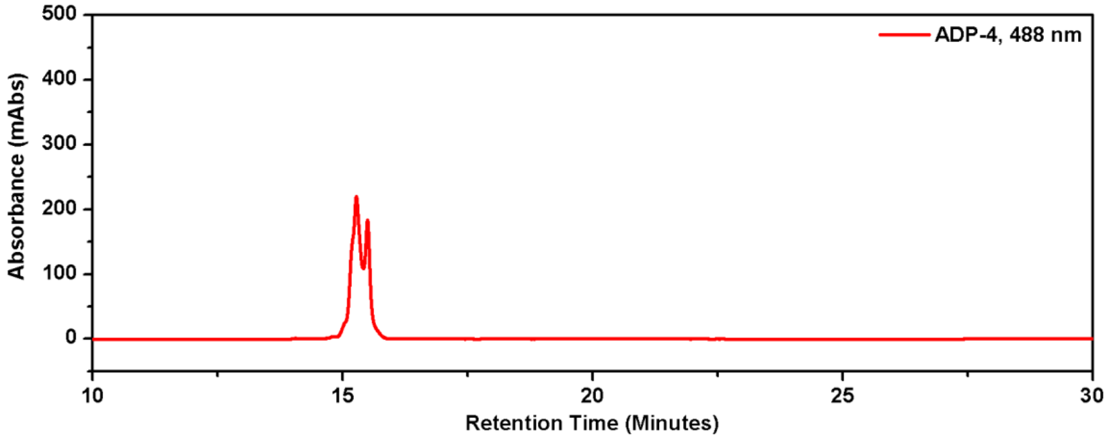

(b)

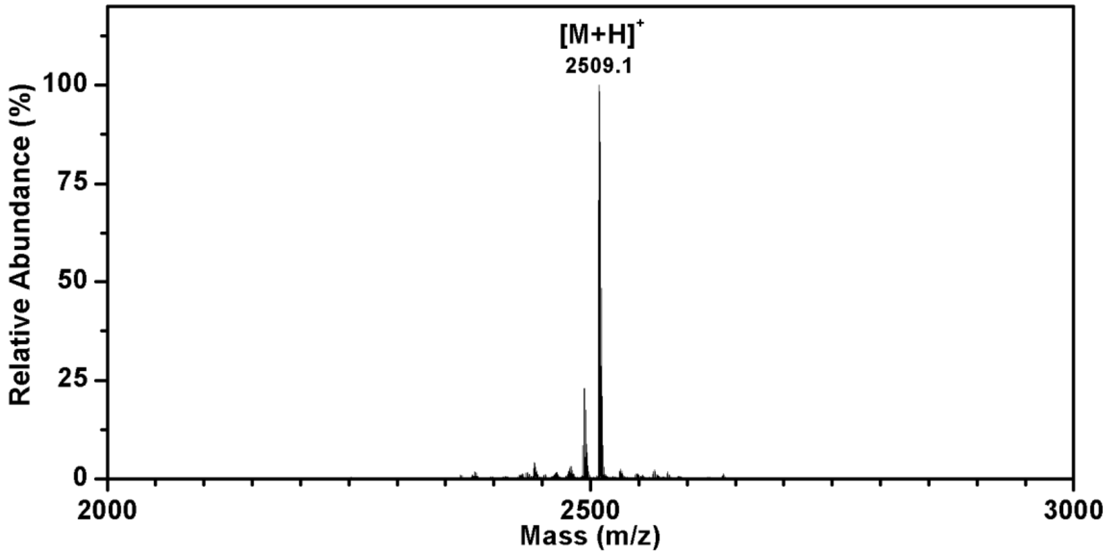

Supplementary Figure 6. HPLC and MS spectra of ADP-4. (a) High performance liquid chromatography spectra of ADP-4 **(b)** Matrix-assisted laser desorption/ionization-time of flight mass spectra of ADP-4, calc. mass: 2507.5; observed: 2509.1 ($[M+H]^+$).

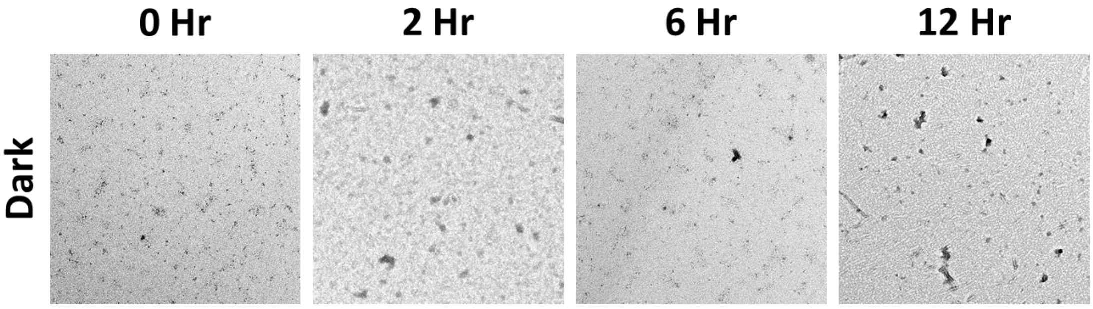

Supplementary Figure 7. TEM images of the incubated ADP-1 without UV irradiation. ADP-1 (50 μM) were prepared in the low salt phosphate buffer saline (0.05 M NaCl, 0.0027 M KCl, 0.01 M Na_2HPO_4, 0.002 M KH_2PO_4, pH = 7.4) and incubated at 37 °C for different time points as indicated. The Dark in the legend indicated the unirradiated condition.

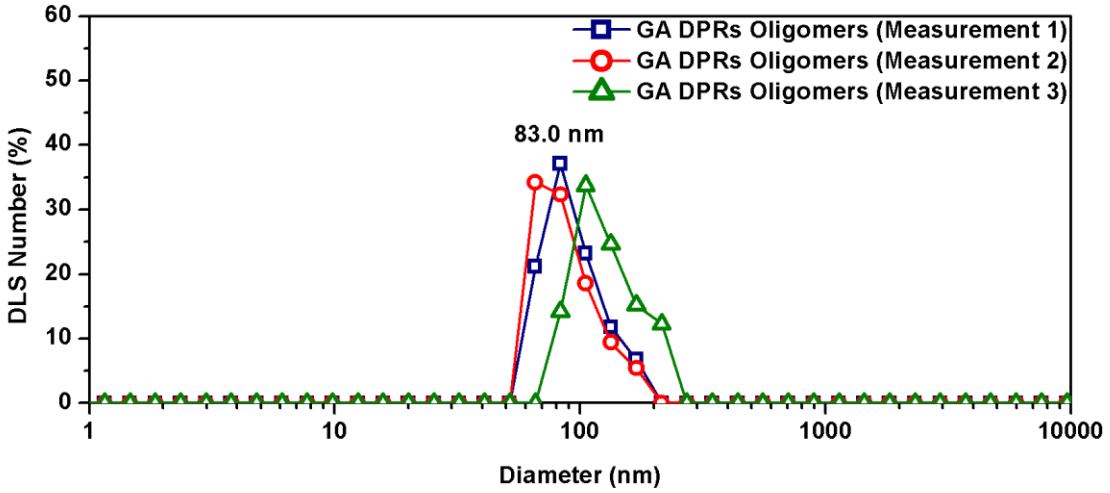

Supplementary Figure 8. Size distribution diagram of ADP-1 oligomers by dynamic light scattering. ADP-1 (100 μM) were irradiated with UV light and incubated for 2 hours before measurements.

(a)

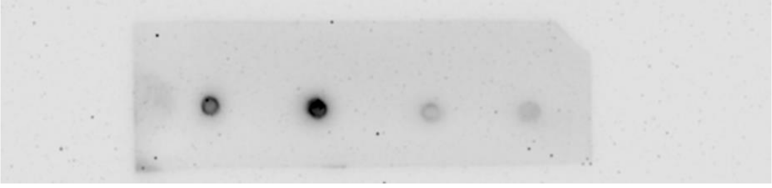

(b)

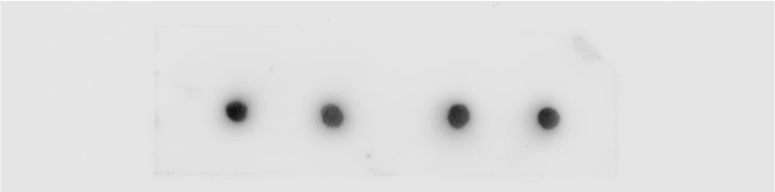

(c)

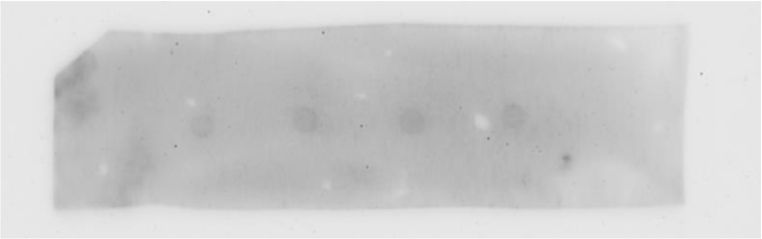

(d)

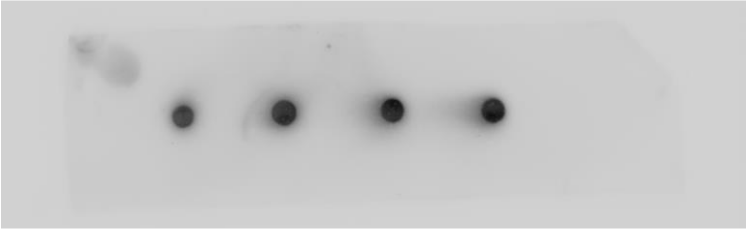

Supplementary Figure 9. Uncropped blot images of ADP-1-induced GA DPRs over time (Figure 1c). The Sample were incubated until the indicated time and applied on membrane. Membranes were stained with anti-GA DPRs antibody and A11 antibody. N=3. **(a)** Photoinitiated ADP-1 stained with A11 antibody. **(b)** Photoinitiated ADP-1 stained with GA DPRs antibody. **(c)** Unirradiated ADP-1 stained with A11 antibody. **(d)** Unirradiated ADP-1 stained with GA DPRs antibody.

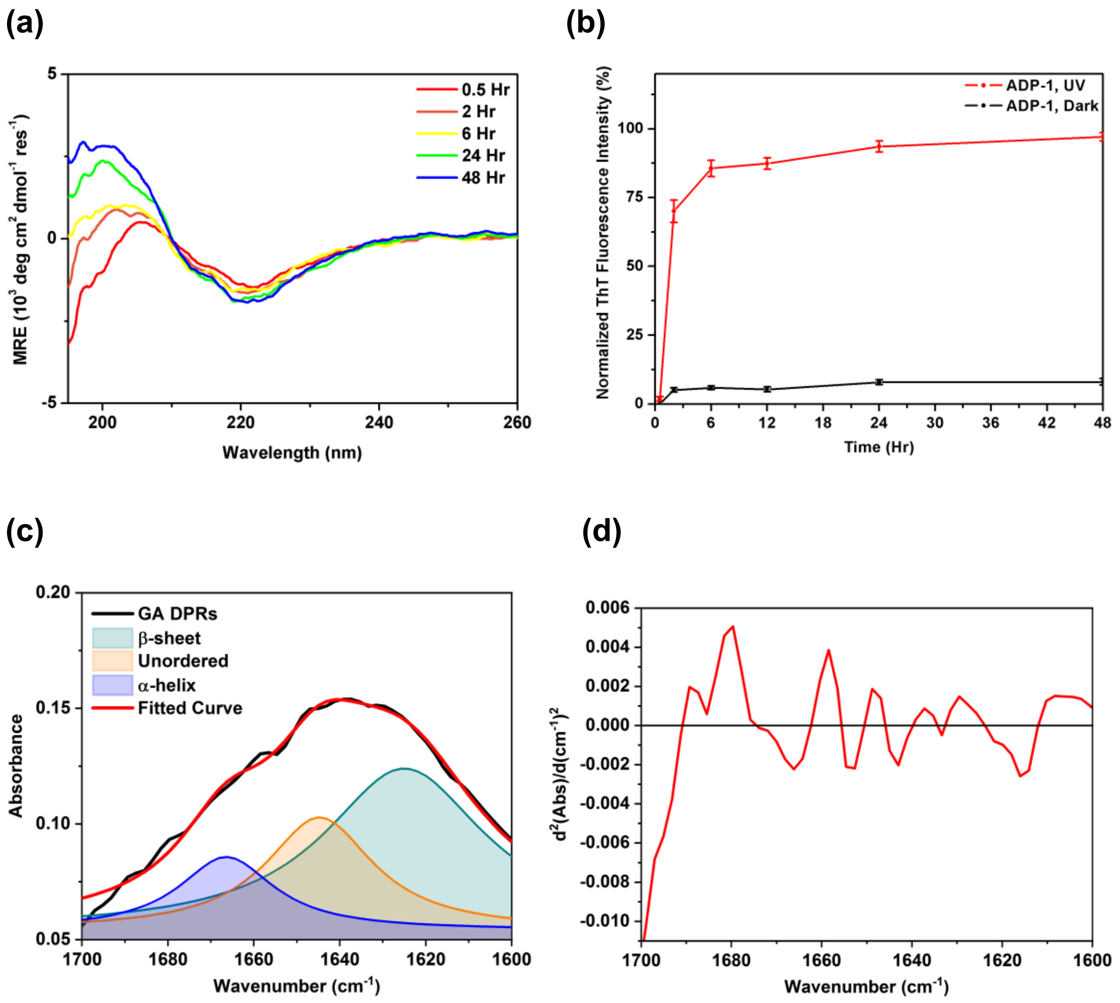

Supplementary Figure 10. Biophysical characterization and thioflavin-binding assay of the GA DPRs from ADP-1. **(a)** Time-course circular dichroism spectrum of photoinitiated ADP-1 (50 μM) in low salt PBS (0.05 M NaCl, 0.0027 M KCl, 0.01 M Na_2HPO_4, 0.002 M KH_2PO_4, pH = 7.4). **(b)** Normalized fluorescence intensity (484 nm) of Thioflavin-T (ThT) mixed with either irradiated or unirradiated ADP-1 (50 μM) in the low-salt PBS. Both ThT and ADP-1 concentration equals to 25 μM in the solution. **(c)** Attenuated total reflectance Fourier-transform infrared spectrum of GA DPRs fibrils. The irradiated and incubated (48 hours) ADP-1 were centrifuged (16000 g) for 30 minutes to collect the GA DPRs fibrils in pellet. Black line indicated the raw IR spectrum of GA DPRs. Red line indicated the fitted curve of GA DPRs IR spectrum. **(d)** Second derivative function of GA DPRs IR spectrum during peak deconvolution.

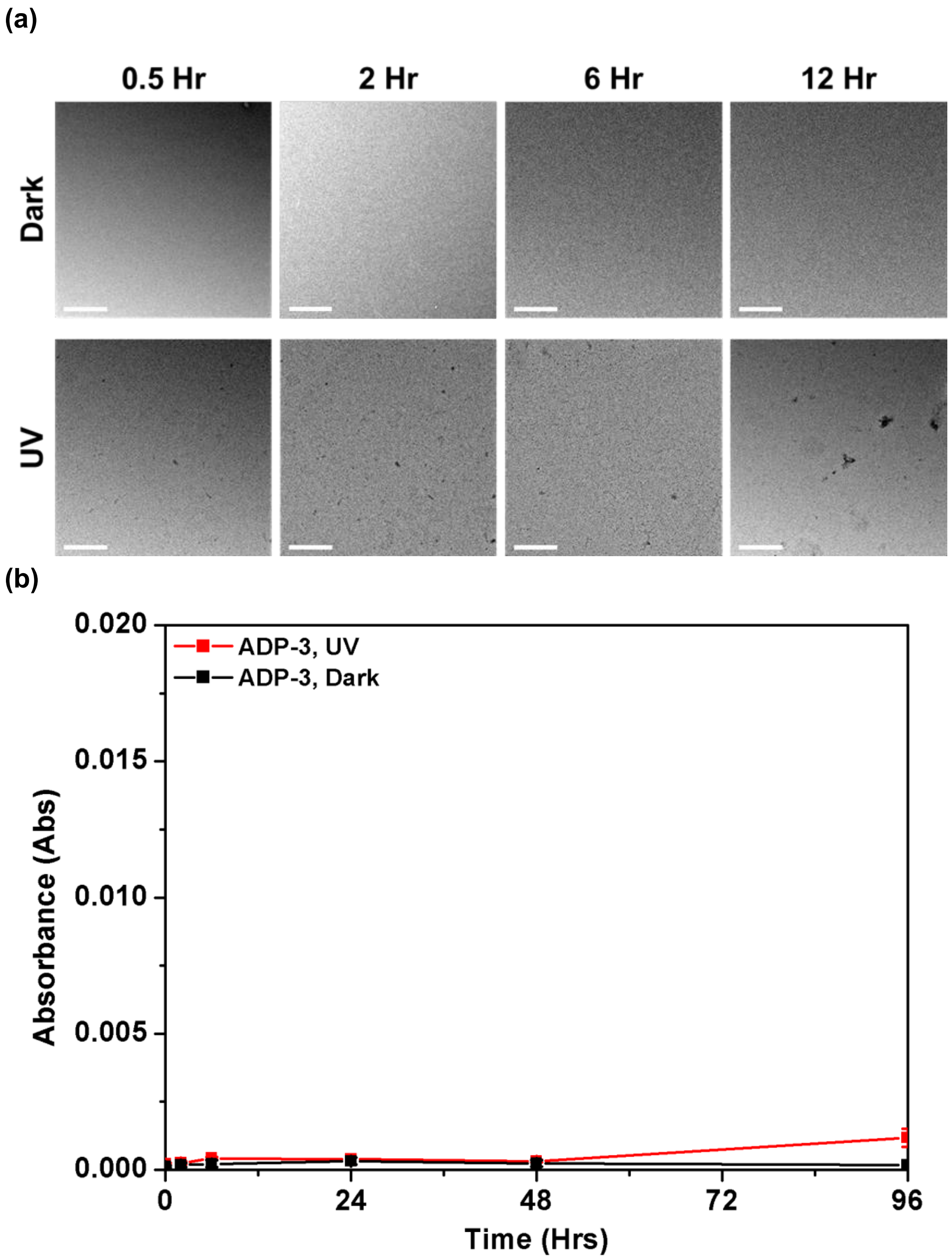

Supplementary Figure 11. Time-course transmission electron microscopy and turbidity measurements of ADP-3. (a) ADP-3 (50 μM) were prepared in the low salt phosphate buffer saline and incubated at 37 °C for different periods. **(b)** ADP-3 (50 μM) were prepared in the low salt phosphate buffer saline either irradiated with UV light or not. The resultant samples were incubated at 37 °C. The Dark in the legend indicated the unirradiated condition.

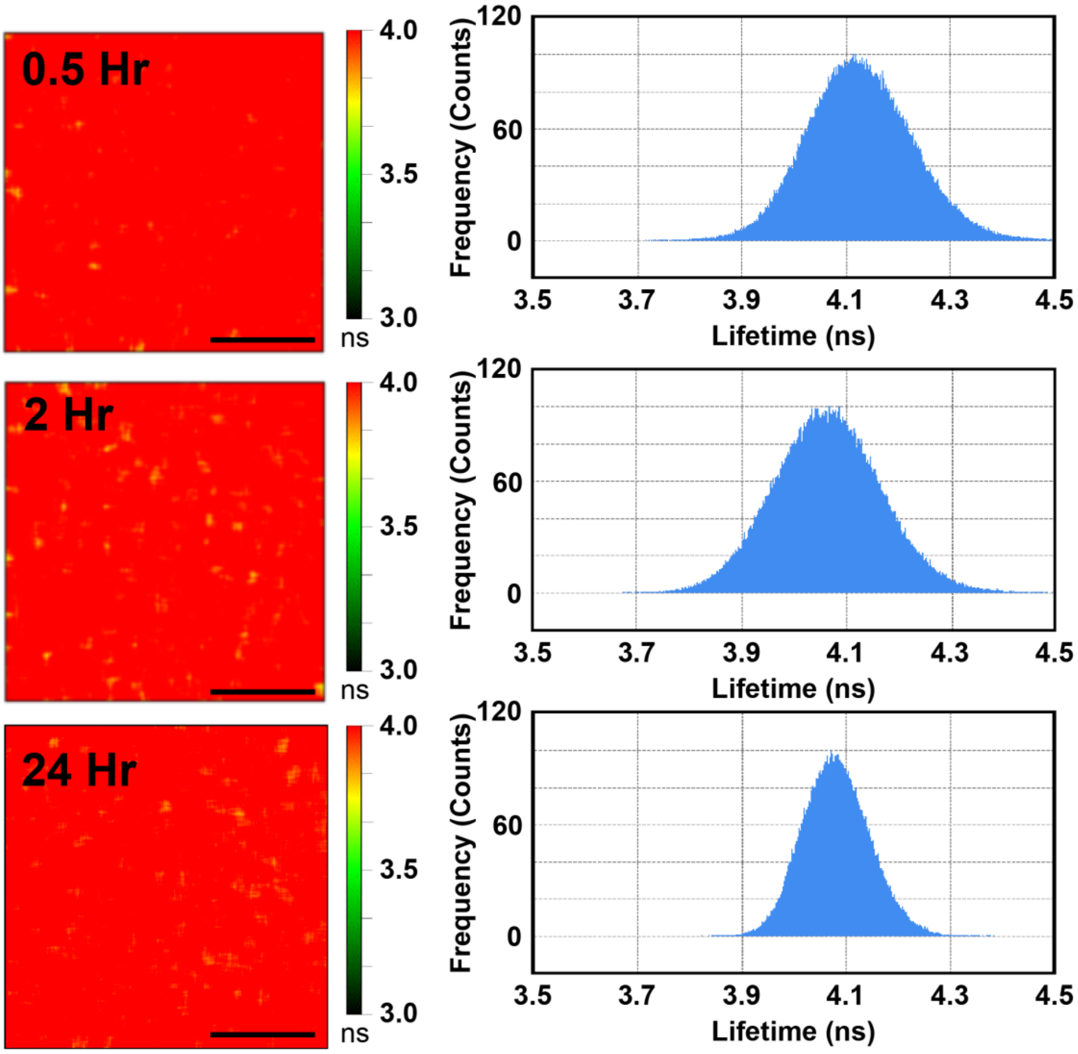

Supplementary Figure 12. Fluorescence-lifetime images and histogram of photointiated ADP-4. ADP-4 (5 μM) were photoinitiated and then incubated to the indicated time. Images were taken at the 0.5th, 2nd, and 24th hour after photoinitiation. Scale bars indicate 5 μm.

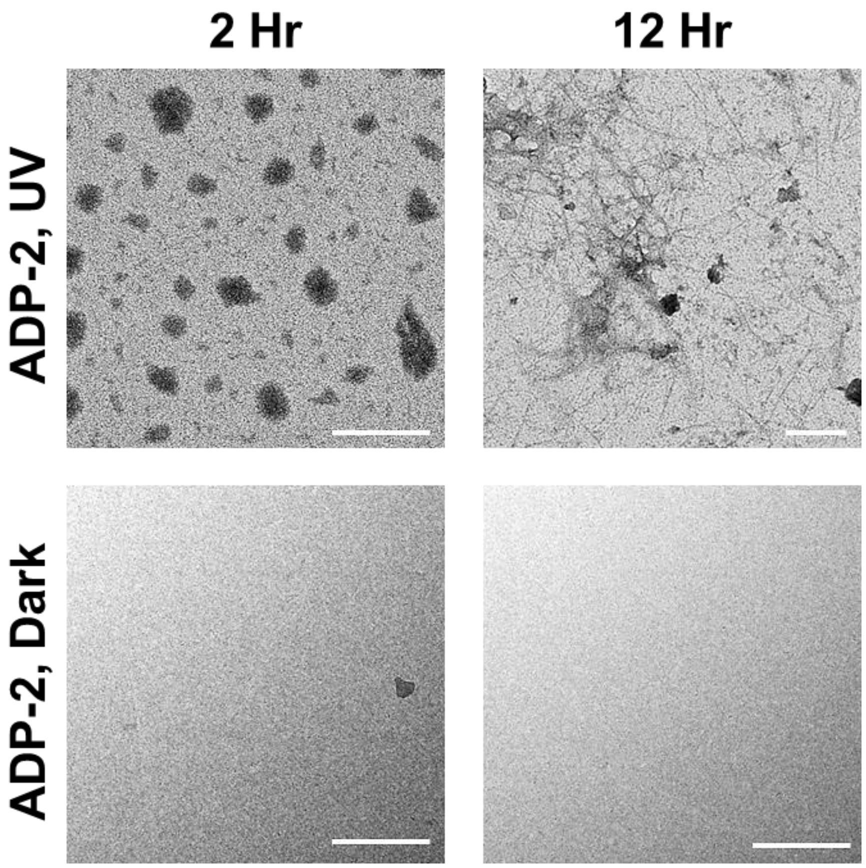

Supplementary Figure 13. Transmission electron microscopy images of ADP-2. ADP-2 (100 μM) were prepared in the low salt phosphate buffer saline and incubated at 37 °C for different periods. Scale bar indicated 100 nm.

(a)

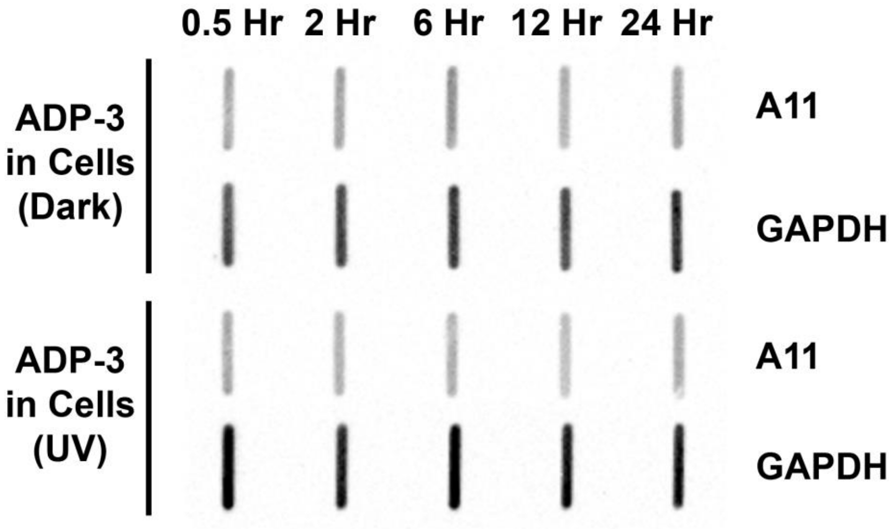

(b)

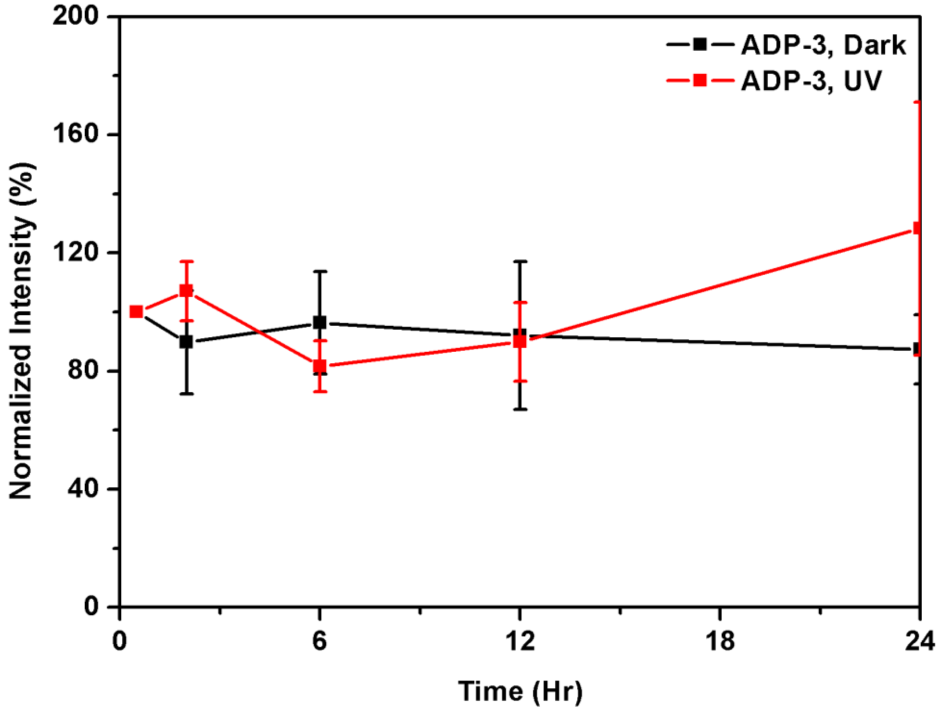

Supplementary Figure 14. A11 immunoblot and quantification analysis on ADP-3 treated SH-SY5Y cell lysate. (a) Immunoblotting of A11 staining on cell lysate from SH-SY5Y cells receiving ADP-3 (1 μM) treatment with either photoinitiation or not. Cell lysates at different time points were harvested and analyzed. **(b)** Quantification analysis of A11 immunoblot of SH-SY5Y cell lysate. The signal of A11 staining was first normalized to that of the GAPDH staining and then compared to signal at 0.5 hour incubation. Three independent experiments were carried out (r = 3).

(a)

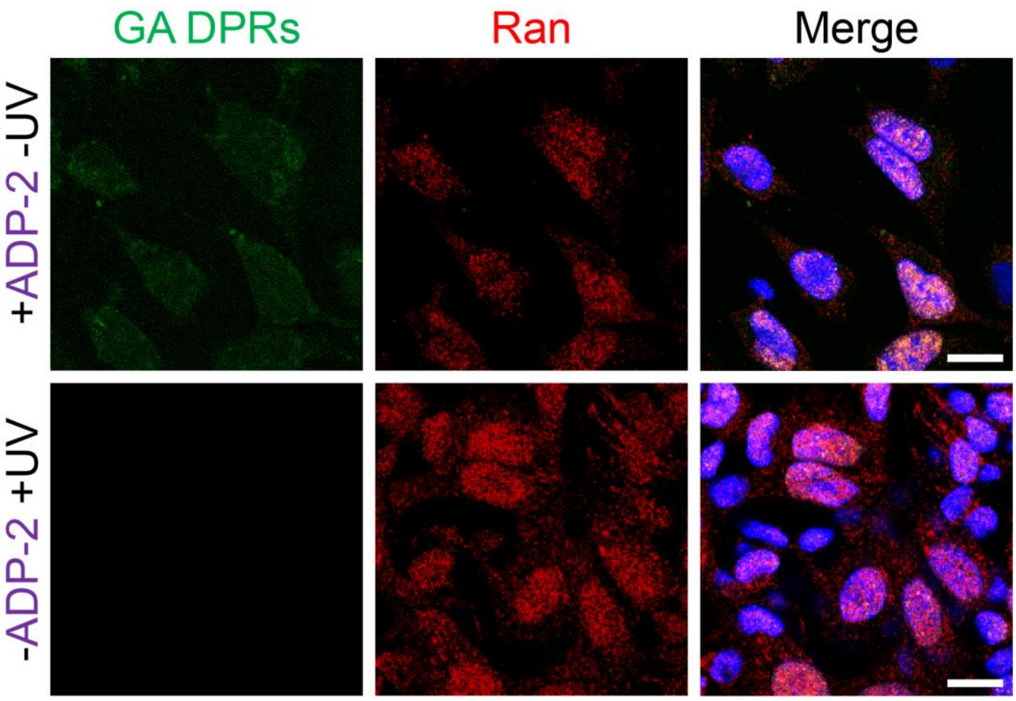

(b)

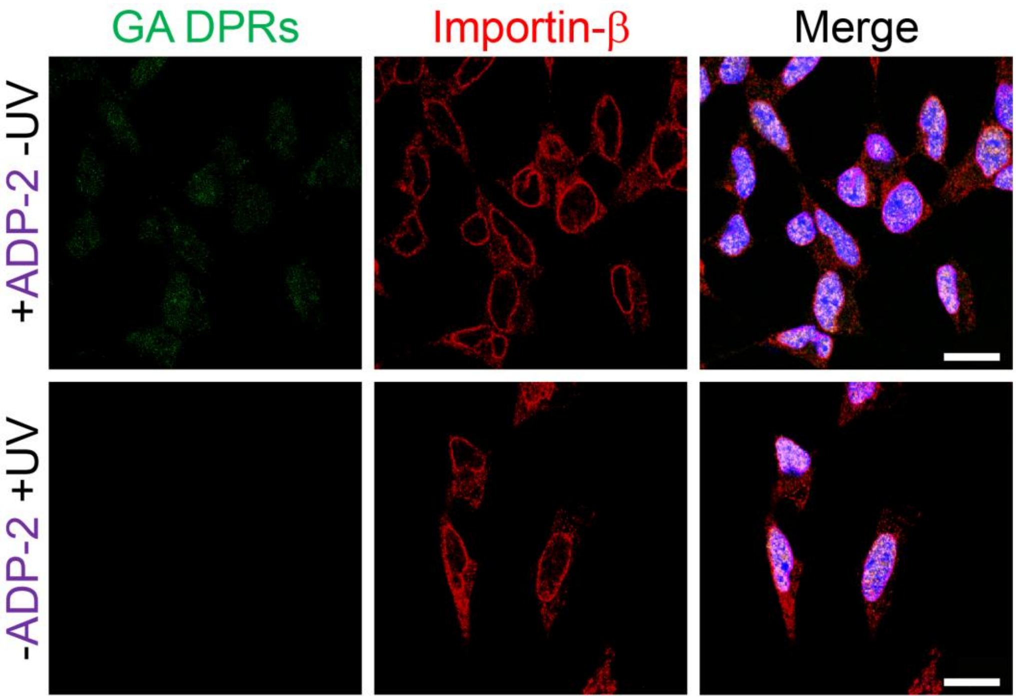

Supplementary Figure 15. Representative images of transport-relevant proteins in SH-SY5Y cells. **(a)** Immunofluorescence images of Ran protein (red) in GA DPRs aggregates (green)-rich SH-SY5Y cells. Cells were treated with ADP-2 (1 μM) and then exposure to UV light. Scale bars indicate 10 μm. **(b)** Immunofluorescence images of Importin-β (red) in SH-SY5Y after ADP-2 (1 μM) and irradiation treatment. Scale bars indicate 10 μm.

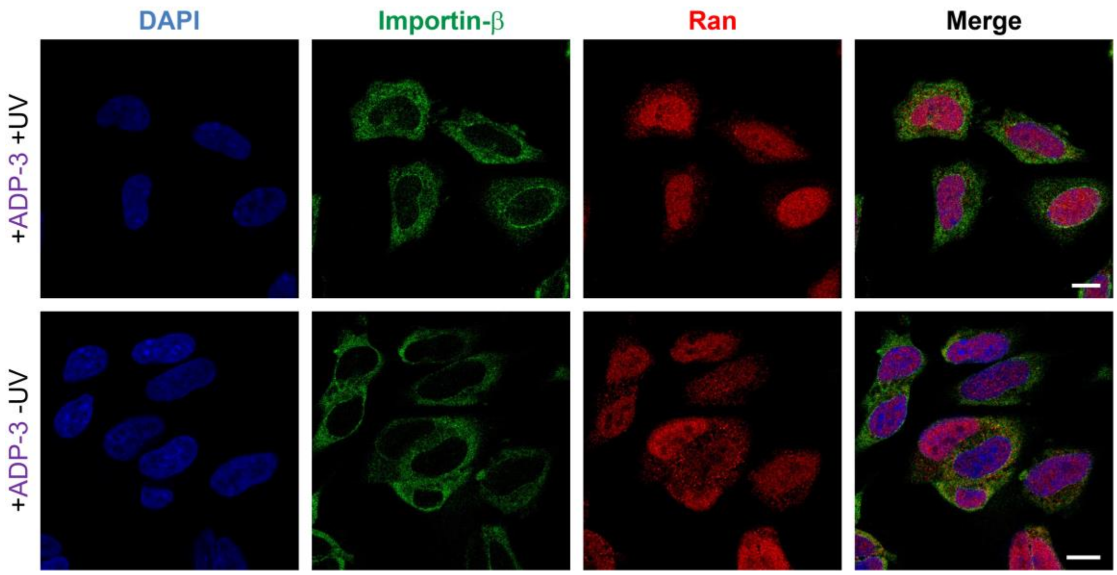

Supplementary Figure 16. **Representative image of Ran protein and importin-β in the SH-SY5Y cells treated with ADP-3.** Immunofluorescence images of Ran protein (red) and importin-β (green) in SH-SY5Y cells. Cells were treated with ADP-3 (1 μM), and then photoinitiated or not. Scale bars indicate 10 μm.

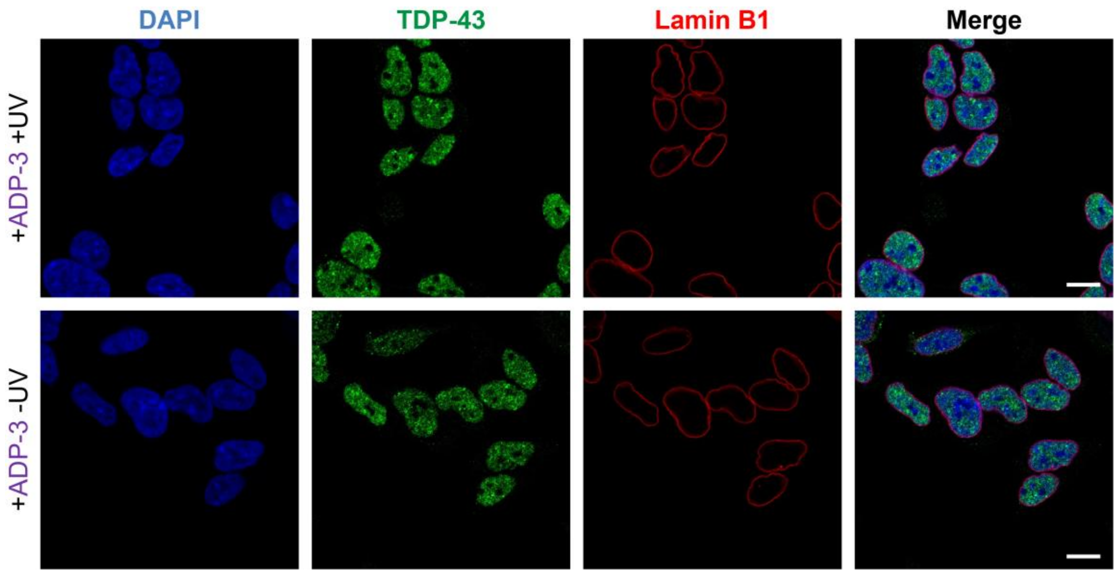

Supplementary Figure 17. **Representative image of TDP-43 and Lamin B1 in the SH-SY5Y cells treated with ADP-3.** Immunofluorescence images of Lamin B1 (red) and TDP-43 (green) in SH-SY5Y cells. Cells were treated with ADP-3 (1 μM), and then photoinitiated or not. Scale bars indicate 10 μm.

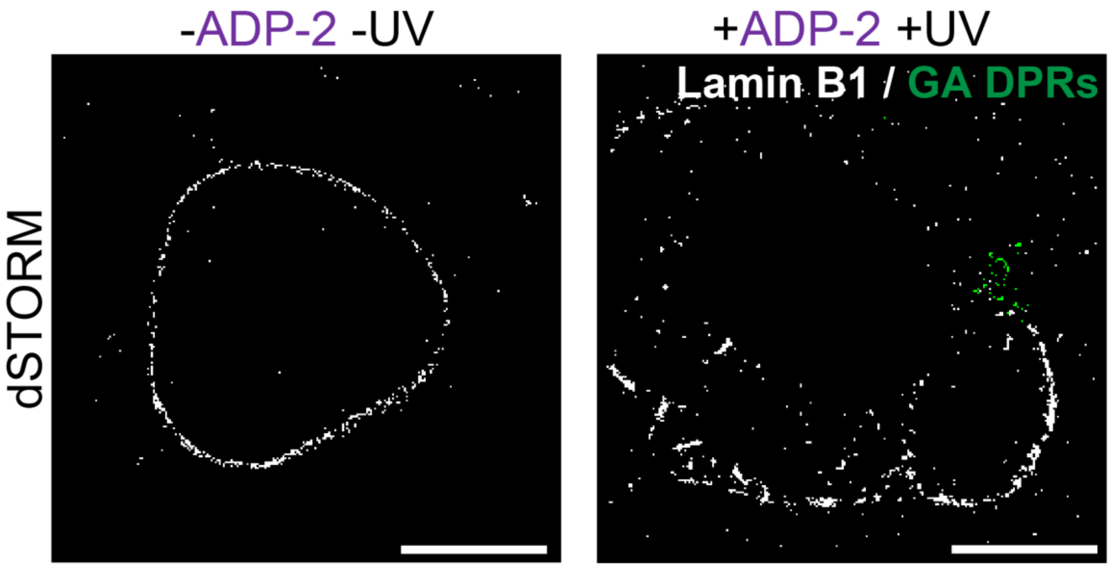

Supplementary Figure 18. Representative dSTORM images of nuclear membrane structure in Cos-7 cells. dSTORM images of lamin B1 (white) and GA DPRs (green) in control or ADP-2 (1 μM) photoinitiated Cos-7 cells. Scale bar indicate 10 μm.

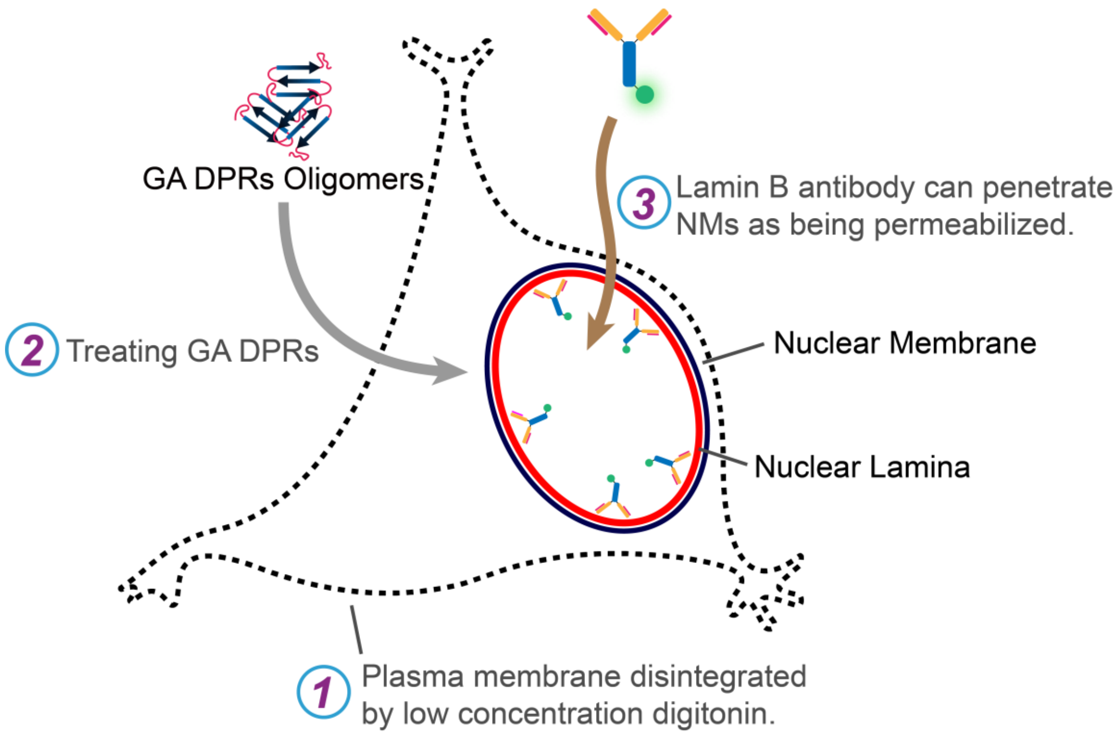

Supplementary Figure 19. Illustration for antibody-accessibility assay experiment. SH-SY5Y cells were first treated with digitonin in PBS to remove their cytoplasmic membrane, followed by GA DPRs treatment for 2 hours. The exposed nuclei were then fixed and stained with lamin B1 antibody and the revealed nuclear lamina structures were observed using confocal microscopy.

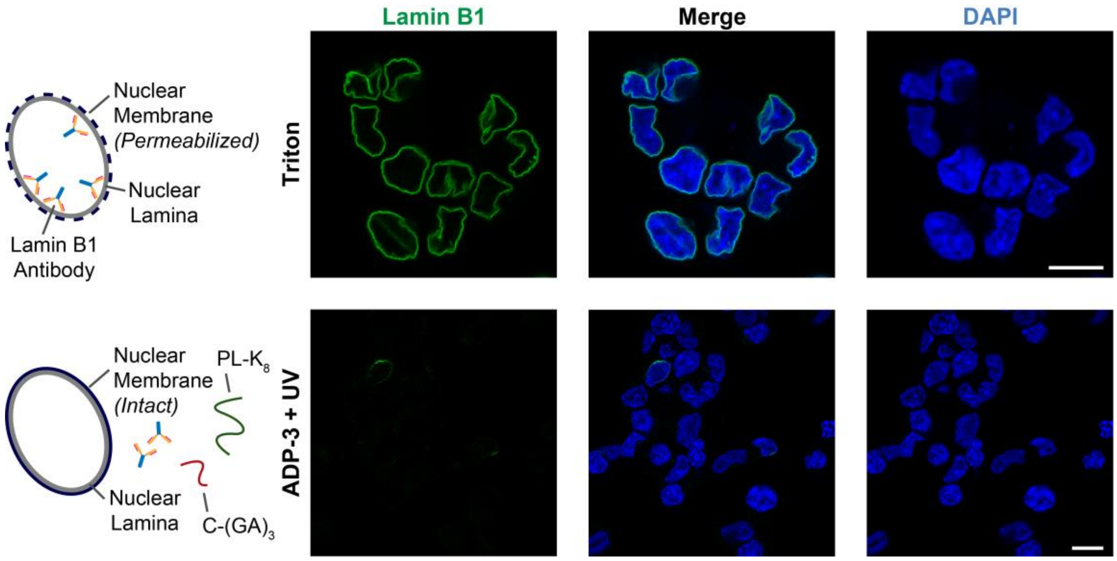

Supplementary Figure 20. Nuclear membrane permeability evaluation using triton and ADP-3 treatment. SH-SY5Y cells were first treated with digitonin in PBS to permeablize their cytoplasmic membrane, followed by triton (positive control) or ADP-3 treatment (negative control). The resulting exposed nuclei were stained with lamin B1 antibody and counterstained with DAPI. The nuclear lamina structures were observed by confocal microscopy. Scale bars indicate 10 µm.

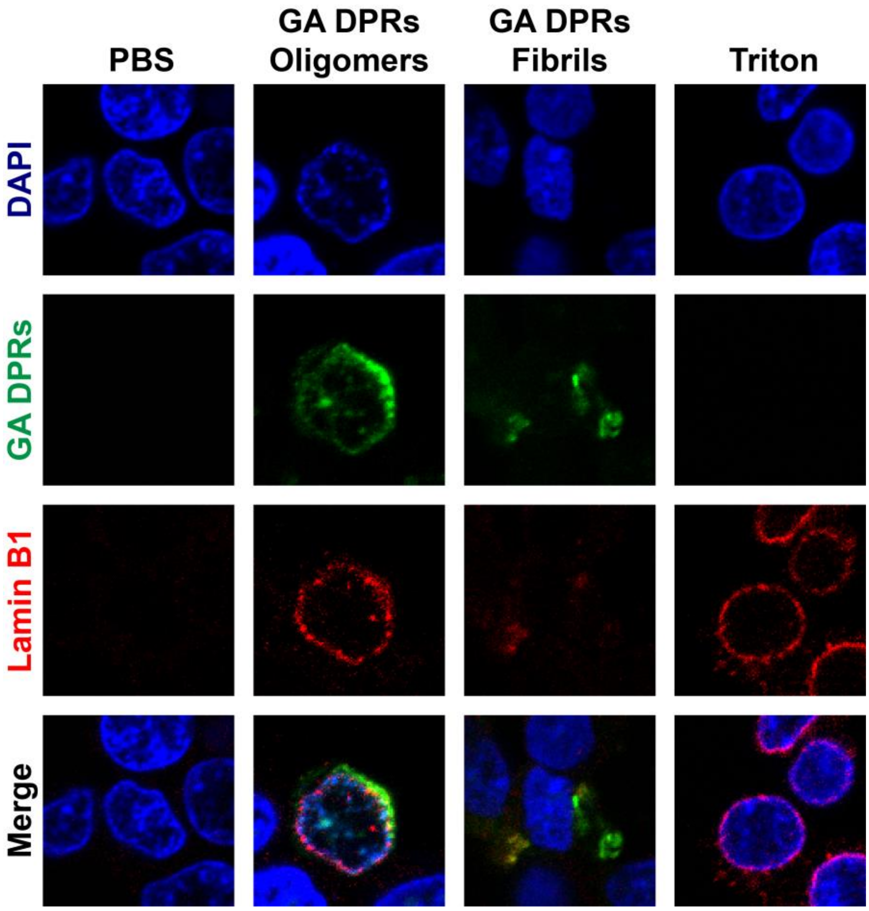

Supplementary Figure 21. Nuclear membrane permeability evaluation using triton and ADP-2-derived GA DPRs oligomers/Fibrils treatment. SH-SY5Y cells were first treated with digitonin in PBS to permeablize their cytoplasmic membrane, followed by triton (positive control) or fluorescent GA DPRs oligomers/fibrils treatment (100 μM, the oligomers/fibrils samples were spiked with ADP-2-derived oligomers/fibrils with ratio equals to 15 %). The resulting exposed nuclei were stained with lamin B1 antibody and counterstained with DAPI. The nuclear lamina structures were observed by confocal microscopy. Scale bars indicate 10 μm.

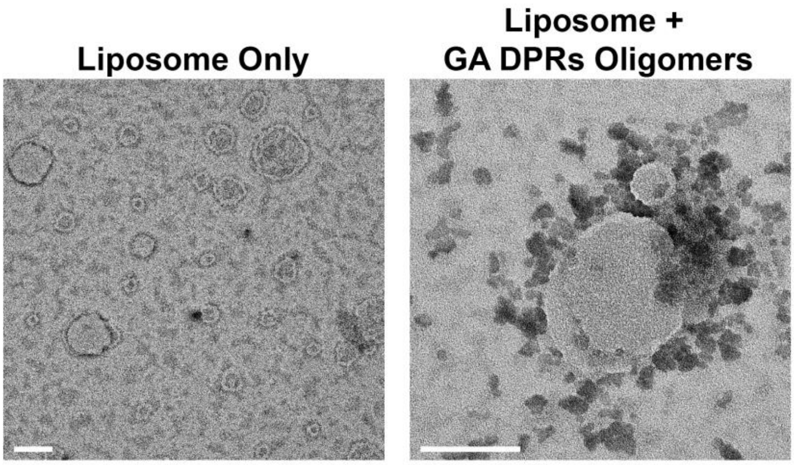

Supplementary Figure 22. Transmission electron microscopy images of lipid liposome interacting with GA DPRs oligomers. The solution containing calcein-encapsulated liposomes were mixed with GA DPRs oligomers (100 µM). The resulting solution were quickly applied on the TEM grids and stained with 1 % phosphotungstic acid. Scale bar indicated 100 nm.

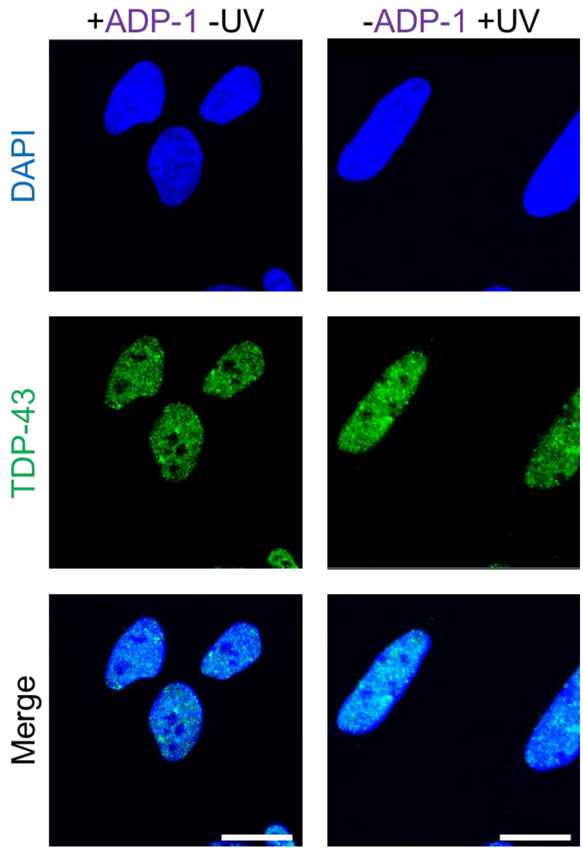

Supplementary Figure 23. Immunofluorescence image of TDP-43 protein in SH-SY5Y cells treated either ADP-1 only or UV light irradiation only. Immunofluorescence of TDP-43 protein (green) in SH-SY5Y cells. Cells were either treated with ADP-1 (1 μM) or exposure to UV light (wavelength: 335-379 nm, power density: ≤ 8.24 mW/cm^2, duration: 1 minute). Scale bars indicate 10 μm.

Supplementary Table

Name	Sequence*	Calc. Mass	Observed Mass
**$(GA)_3$**	$(GA)_3$-NH_2	401.4	424.2 $(M+Na)^+$
**$(GA)_{12}$**	$(GA)_{12}$-NH_2	1554.6	1576.8 $(M+Na)^+$
**$(GA)_{20}$**	$(GA)_{20}$-NH_2	2578.2	2601.5 $(M+Na)^+$
**ADP-1**	C-$(GA)_{12}$-#PL-K_8-NH_2	2963.4	2963.7
**ADP-2**	AF 488 C5 Maleimide-C-$(GA)_{12}$-#PL-K_8-NH_2	3661.0	3661.2
**ADP-3**	C-$(GA)_3$-#PL-K_8-NH_2	1809.9	1811.0
**ADP-4**	AF 488 C5 Maleimide-C-$(GA)_3$-#PL-K_8-NH_2	2507.5	2509.1 $(M+H)^+$

Supplementary Table 1. List of peptides, sequence, and mass of the peptide used in this work. *Sequence was written in the direction from N-terminus to C-terminus. #PL stands for the photolabile linker.

Supplementary Methods

Turbidity measurement, circular dichroism, infrared spectroscopy and dye-binding assay

Peptide solution containing ADP-1 (50 μM) or ADP-3 (50 μM), were prepared in low salt PBS (0.05 M NaCl, 0.0027 M KCl, 0.01 M Na_2HPO_4, 0.002 M KH_2PO_4, pH = 7.4) either irradiated with UV light (365nm, power density: 32 mW/cm^2, duration: 1 min) or not. Peptide containing solutions were incubated at 37 °C for following measurements.

The turbidity of ADP-1 and ADP-3 solutions was recorded with a 1 cm quartz cuvette on the DU800 spectrophotometer (Beckman, U.S.A.). All turbidity data were collected at 600 nm. The CD spectra of the resulting $(GA)_{12}$ solutions from ADP-1 were measured with a 1 mm quartz cuvette on the J-815 CD spectrometer (JASCO, Japan). All data were collected from 195 to 260 nm with a scanning speed of 200 nm/min. Ten scans were averaged for each sample.

For infrared spectroscopy measurements, the photoinitiated ADP-1 was incubated for 48 hours at 37 °C, and then centrifuged at 16000 g for 30 minutes to collect the pellet. The resulting pellet was washed with ethanol and then dried in vaccum for overnight. The attenuated total reflectance fourier-transform infrared spectrometer (FT/IR-6700, Jasco, Japan) was used to measure the infrared specrum of GA DPRs. The deconvolution of IR spectrum was achieved by Origin 2021 with Peak Deconvolution App. The Lorentzian spectrrum were deconvoluted by second derivative method and smoothed by Savitzky Golay with 2 polynomial order and 9 points of window.

For thioflavin T binding assay, ThT working solution (50 μM ThT in PBS) was freshly prepared and filtered through a 0.22 μm Millipore filter. A 40 μL aliquot of the ADP-1 solution was mixed with 40 μL of the ThT working solution for 5 min at room temperature. The fluorescence emission spectra at 480 nm were taken in a 3 mm path length rectangular fluorescence quartz cuvette on an F-4500 spectrofluorimeter (Hitachi, Japan) with excitation at 442 nm.

Transmission electron microscopy

For peptide TEM analysis, ADP-1, ADP-3, $(GA)_3$, $(GA)_{12}$ and

$(GA)_{20}$ (50 μM) were prepared in low salt PBS buffer (0.05 M NaCl, 0.0027 M KCl, 0.01 M Na_2HPO_4, 0.002M KH_2PO_4, pH = 7.4) irradiated with or without UV, respectively (365 nm, 1200 μw/cm^2, duration: 1 min) followed by 37 °C incubation at the indicated time points. The resulting aliquots of peptide solutions (5 μL) were applied on glow-charged 300 mesh Formvar- and carbon-coated copper grids and stained with 1 % uranyl acetate. After drying overnight, all samples were analyzed using a JEM-2011 electron microscope (JEOL, Japan).

For the liposome TEM imaging, fresh prepared liposomes encapsulated with calcein were mixed with 100 μM with GA DPRs oligomers. The resulting solution (5 μL) were quickly applied on the n glow-charged 300 mesh Formvar- and carbon-coated copper grids and stained with 1 % phosphotungstic acid. After drying overnight, all samples were analyzed using a JEM-2011 electron microscope (JEOL, Japan).

For cellular TEM imaging , cos-7 cells treated with ADP-1 and irradiated were fixed by 4 % glutaraldehyde in PBS. Cell membrane of cos-7 was stained with osmium tetroxide (1 % in PBS). After cell dehydration with alcohol, cell samples were ultracut using diamond knife to acquire thin section of cell. The thin cell sections were then put on the grid and stained with 1 % uranyl acetate. After drying overnight, all samples were analyzed using a JEM-2011 electron microscope (JEOL, Japan).

Dot blot analysis

ADP-1 and ADP-3 (100 μM) were freshly prepared in low salt PBS buffer (0.05 M NaCl, 0.0027 M KCl, 0.01 M Na_2HPO_4, 0.002 M KH_2PO_4, pH = 7.4) irradiated with or without UV (365 nm, 1200 μw/cm^2, duration: 1 min), and then incubated at 37 °C until the time indicated. 2 μL aliquot of incubated solution was applied on the nitrocellulose blotting membrane. membrane was dried, blocked with 5 % bovine serum albumin in tris-buffer saline with 0.1 % Tween 20, and stained with A11 antibody (AHB0052, ThermoFisher) and GA repeat antibody (24492-1-AP, Proteintech), respectively.

Direct stochastic optical reconstruction microscopy

dSTORM images were captured with ELYRA superresolution

microscope (Zeiss). For an appropriate blinking buffer condition of thiol reduction for dSTORM, mercaptoethylamine and 2-mercaptoethanol at 75 mM were used for optimized blinking duty cycles and duration. Glucose oxidase (160 mg/mL) and catalase (0.5 mg/mL) was used to prevent photolysis of excited dyes. All the dSTORM imaging was performed with an Elyra PS.1 superresd into imaging buffer combining 10 % of glucose to scavenge oxygen and avoid oxygen-induced solution microscope. The region of interest (ROI) was selected with a 100x, NA 1.46, plan-APO objective. An EMCCD Andor iXon 897 (pixel size 16 μm; Optovar lens, magnification 1.6x, along with the 100x objective lens yielded a final pixel size of 100 nm) was used as the camera detector. With laser illumination at 488 nm and an emission filter was matched to A488 emission spectrum, 50000 raw images of blinking molecules were acquired with EPI or TIRF microscopy mode. The dSTORM images were reconstructed with a pixel size of 10 nm. For the cellular images, SH-SY5Y and Cos-7 were cultured, treated with ADP-2 and photoinitiated as aforementioned. Cells were fixed and stained with corresponding antibodies as indicated. The Cell samples were immersed in the blinking buffer and 50000 raw images of blinking molecules were collected and reconstructed to acquired dSTORM images as described above.

Cell maintenance, peptide treatment, and photoinitiation

Human neuroblastoma SH-SY5Y (provided by Dr. Wen Zhi-Hong, Department of Marine Biotechnology and Resource, National Sun Yat-sen University) was maintained in DMEM/F12 medium and monkey kidney Cos-7 (provided by Dr. Pang-Hsien Tu, Institute of Biomedical Sciences, Academia Sinica) cells were cultured in Dulbecco's modified Eagle's medium (Invitrogen) respectively supplemented with 2 mM glutamine, 10 % heat-inactivated fetal bovine serum, and 100 U/mL penicillin−streptomycin (Invitrogen) at 37 °C in a humidified atmosphere with 5 % CO_2. These cell lines were not tested by PCR; however, DAPI staining confirmed little or none Mycroplasma. Peptides (ADP-1, ADP-2, ADP-3, ADP-4) solution were prepared by dissolving the lyophilized powder in fresh cell culture medium. To photoinitiate ADP peptides in cells, cells were pre-loaded with polypeptide-containing medium for 6 h,

washed, and UV-illuminated (mercury lamp with 345−385 nm bandpass filter; average power: 8.24 mW/cm^2; duration: 1 min). After the photoinitation, the cells will be washed with fresh medium for three times to remove the extracellular peptides. Cells will be further incubated for 24 hours and then fixed by 4 % paraformaldehyde for imaging observation.

Dynamic light scattering measurements

To measure the size distribution of GA DPRs oligomers, ADP-1 (100 μM in low salt PBS buffer was UV-irradiated followed by 2 hours incubation at 37 °C using DynaPro NanoStar (Wyatt technology, U.S.A.). Samples were measured at 25 °C, and more than 15 times measures were averaged to acquire correlation function curve. The correlation function curve was later fitted with regularization fitting. The results with sum of squares (SOS) larger than 100 will be excluded.

Immunostaining and confocal microscopy

For TDP-43, Ran, Importin-β and Lamin B1 immunohistochemistry experiments, 2 × 10^5 of SH-SY5Y or Cos-7 cells on a 30 mm square coverslip were treated with ADP-1, 2, or 3 peptides basically as described above. The photoinitiated cells were incubated for an additional 24 hrs and then fixed with 4 % paraformaldehyde. For immunostaining, anti-TDP-43 antibody (Abcam, ab104223), anti-Ran antibody (Abcam, ab155103), anti-importin-β antibody (Abcam, ab2811), anti-Lamin B1 antibody (Abcam, ab16048) and anti-Nup153 antibody (Abcam, ab24700) were used respectively. The coverslips with the immunostained cells were sealed using mounting media (Invitrogen, ProLong™ Diamond Antifade Mountant). Confocal images of the resulting samples were captured with LSM 780 & LSM 880 (Zeiss). All images were captured under blind condition.

TIRF imaging for quantitation of sGFP distribution

TIRF and epifluorescence images were collected using a Nikon TiE microscope, where shuttling GFP (sGFP) were excitated by 488nm filter set with 130w mercury lamp. For transfection of sGFP on SH-SY5Y cells, 1.5 ug of DNA (NES-eGFP-NLS) were mixed

with lipofectamine 3000 reagents according to manufacturer's instruction (ThermoFisher) and then added to the 5 × 10^5 of SH-SY5Y cells on the 30 mm square coverslip. After 24 hours, cells were treated with peptides and illumination according to aforementioned descriptions.

ADP-1 treatment in mouse cortical neurons, immunofluorescence staining, and neurite fragmentation and TDP-43 nuclear to cytoplasmic ratio analysis

All animal experimental procedures were approved by the Institutional Animal Care and Use Committee (IACUC) and in accordance with the Guide for the Care and Use of Laboratory Animals of National Chiao Tung University. Primary culture of dissociated mouse cortical neurons isolated from the brains of E17.5 (embryonic 17.5) mouse pups was carried out as previously described.[1] The ADP-1 peptide (final concentration 1 μM) was dissolved in the pre-equilibrated B27-supplemented neurobasal medium containing 50 % of the old cultured medium. The 21 days in vitro (21DIV) cortical neurons were incubated with null or ADP-1 -containing medium for 8 hours. To completely remove ADP-1 from the culture medium, the washing process, involving replacement with 500 μL of pre-equilibrated B27-supplemented neurobasal medium, was applied once. Cortical neurons were then UV irradiated for 2 minutes using a Nikon Intensilight epi-fluorescence light source, a Semrock filter FF01-357/44-25, and a 10x 0.45 N.A. Plan Apochromat objective lens. 24 hours after UV irradiation, cortical neurons were fixed with 3.7 % formaldehyde in 1× PBS at 37 °C for 15 min, followed by membrane permeabilization with 0.25 % Triton X-100 for 5 min at room temperature. Cells were then blocked with 10 % bovine serum albumin (BSA) in 1× PBS for 30 min at 37 °C followed by 1 hour of incubation at 37 °C with primary antibodies (anti-TDP-43, 1:1000, Abcam, ab104223; anti-β-III-tubulin, 1:200, Abcam, ab18207) in 2% BSA. Cells were washed 3 times with 1× PBS and subjected to 1 hour of incubation with AlexaFluor 488-labeled (anti-mouse) and 568-labeled (anti-rabbit) secondary antibodies (1:1000, Thermo Fisher Scientific) at 37 °C in the dark. Immunofluorescence stained neurons were acquired on a Nikon Eclipse-Ti inverted microscope equipped with a 60× 1.49 N.A.

Plan Apochromat objective lens, an Intensilight epi-fluorescence light source, a Photometrics CoolSNAP HQ2 camera, and Nikon NIS-Elements software 4.13.05. To quantify the degeneration area of neurites, ImageJ (http://imagej.nih.gov/ij/) was used to process and analyze the neurite signal in the β-III-tubulin channel. Briefly, the total neurite area was obtained from the original β-III-tubulin image using Subtract Background → Gaussian Blur → Phansalkar auto local threshold. The fragmented neurite area was obtained using the analyze particles function with a particle size less than 50 pixels. The degeneration area percentage (%) of neuron is quantified as fragemnted neurite area / total nerutite area.

For the analysis of TDP-43 nuclear to cytoplasmic ratio in neurons, we first used the fluorescence signal from TUJ1 staining to manually select the total soma area. The DAPI staining was used to select the nuclear area. The cytoplasmic area was selected by subtracting the nuclear area from the soma area. The TDP-43 intensity in nuclei was quantified from the signal in nuclear area, and the TDP-43 intensity in cytoplasm was then quatified from the signal in cytoplasmic area. The nuclear to cytoplasmic ratio was achieved by dividing the fluorescence intentisty of nuclear TDP-43 over fluorescence intentisty of cytoplasmic TDP-43 (fluorescence intentisty of nuclear TDP-43 / fluorescence intentisty of cytoplasmic TDP-43). More than 50 neurons were counted from three individual experiments (r = 3).

Imaging Quantification and Statistical Analysis.

To evaluate the ratio of nuclear Ran depletion in treated or untreated SH-SY5Y cells, the fluroescence intensity from Ran antibody immunohistochemistry in cytoplasm and nucleus were analyzed by ImageJ. The fluorescence intensity in nucleus was acquired by isolating the nuclear area through overlapping with DAPI staining, while the fluorescence intensity in cytoplasm was acquired by subtracting the total fluorescence intensity with the fluorescence intensity in nucleus. Once the fluorescence intensity in cytoplasm was three times higher than fluorescence intensity in nucleus, that cell was determined as nuclear Ran depletion.

To evaluate the ratio of importin-β diffusion in treated or untreated SH-SY5Y cells, the fluroescence intensity from importin-

β antibody immunohistochemistry in cytoplasm and perinucleus region were analyzed by ImageJ. The cell was determined as cell with importin-β diffusion if the integrated fluorescence intensity in the cytoplasm is two times higher than the integrated fluorescence intensity in the perinucleus region via fluorescence intensity profiling.

The quantification of sGFP distribution was carried out using ImageJ for evaluation flourescence intensity in cytoplasm and nucleus. The sGFP fluorescence intensity in nucleus was acquired by isolating the nuclear area through overlapping with DAPI staining channel, while the sGFP fluorescence intensity in cytoplasm was acquired by subtracting the total fluorescence intensity with the fluorescence intensity in nucleus. The resulting nuclear-to-cytoplasmic ratio of sGFP for each cells was acquired by fluorescence intensity in nucleus over the fluorescence intensity in cytoplasm.

The quantification of nuclear diffusion of Lamin B1 in treated or untreated SH-SY5Y cells was achieved by fluorescence intensity profiling using ImageJ. The cell was determined as cell with Lamin B1 staining in nucleus if the integrated fluorescence intensity in the nucleus is higher than the integrated fluorescence intensity in the perinucleus region via fluorescence intensity profiling.

To evaluate the antibody penetrance level, ImageJ was employed. The nuclear remains will be determined as Lamin B1 staining positive if the fluorescence signals in perinuclear region were noticed.

The cytosolic retention ratio of TDP-43 in SH-SH5Y cells was carried out by analyzing the fluorescence distribution of TDP-43 antibody immunohistochemistry in cytoplasm and nucleus using ImageJ. The fluorescence intensity in nucleus was acquired by isolating the nuclear area through overlapping with DAPI staining channel, while the fluorescence intensity in cytoplasm was acquired by subtracting the total fluorescence intensity with the fluorescence intensity in nucleus. The cell will be determined as TDP-43 cytosolic retention positive if the fluorescence intensity in cytoplasm is higher than 20% of total fluorescence.

All experimental comparisons were analyzed using two-sided Welch’s T test. The Bonferroni correction was used for multiple comparison correction.

Figures were exported through Zen 3.1 (Carl Zeiss, Germany) and OriginPro 2021 (OriginLab, U.S.A.).

Supplementary Reference

1 Chen, W.-S. *et al.* Ran-dependent TPX2 activation promotes acentrosomal microtubule nucleation in neurons. *Sci. Rep.* **7**, 42297, doi:10.1038/srep42297 (2017).
