# Supplementary material for: Nanoscopic investigation of C9orf72 poly-GA oligomers on nuclear membrane disruption by a photoinducible platform
Source: Commun Chem. 2021 Jul 23;4:111. doi: 10.1038/s42004-021-00547-6 (PMC9814621; doi:10.1038/s42004-021-00547-6)
Supplement: Supplementary file 4 — Reporting Summary [file 42004_2021_547_MOESM4_ESM.pdf]

nature research

Corresponding author(s): Jen-Tse Huang

Last updated by author(s): Jun 21, 2021

Reporting Summary

Nature Research wishes to improve the reproducibility of the work that we publish. This form provides structure for consistency and transparency in reporting. For further information on Nature Research policies, see our Editorial Policies and the Editorial Policy Checklist.

Statistics

For all statistical analyses, confirm that the following items are present in the figure legend, table legend, main text, or Methods section.

n/a	Confirmed	
☐	☒	The exact sample size (n) for each experimental group/condition, given as a discrete number and unit of measurement
☐	☒	A statement on whether measurements were taken from distinct samples or whether the same sample was measured repeatedly
☐	☒	The statistical test(s) used AND whether they are one- or two-sided<br>*Only common tests should be described solely by name; describe more complex techniques in the Methods section.*
☐	☒	A description of all covariates tested
☐	☒	A description of any assumptions or corrections, such as tests of normality and adjustment for multiple comparisons
☐	☒	A full description of the statistical parameters including central tendency (e.g. means) or other basic estimates (e.g. regression coefficient) AND variation (e.g. standard deviation) or associated estimates of uncertainty (e.g. confidence intervals)
☐	☒	For null hypothesis testing, the test statistic (e.g. F, t, r) with confidence intervals, effect sizes, degrees of freedom and P value noted<br>*Give P values as exact values whenever suitable.*
☒	☐	For Bayesian analysis, information on the choice of priors and Markov chain Monte Carlo settings
☒	☐	For hierarchical and complex designs, identification of the appropriate level for tests and full reporting of outcomes
☒	☐	Estimates of effect sizes (e.g. Cohen's d, Pearson's r), indicating how they were calculated

Our web collection on statistics for biologists contains articles on many of the points above.

Software and code

Policy information about availability of computer code

Data collection	No software was used
Data analysis	Zen, Origin, Fiji

For manuscripts utilizing custom algorithms or software that are central to the research but not yet described in published literature, software must be made available to editors and reviewers. We strongly encourage code deposition in a community repository (e.g. GitHub). See the Nature Research guidelines for submitting code & software for further information.

Data

Policy information about availability of data

All manuscripts must include a data availability statement. This statement should provide the following information, where applicable:

- Accession codes, unique identifiers, or web links for publicly available datasets
- A list of figures that have associated raw data
- A description of any restrictions on data availability

The authors declare that all data supporting the findings of this study are available within the paper and its supplementary information files (Supplementary Information.docx / Statistical Analysis.xlsx).

Field-specific reporting

Please select the one below that is the best fit for your research. If you are not sure, read the appropriate sections before making your selection.

☒ Life sciences ☐ Behavioural & social sciences ☐ Ecological, evolutionary & environmental sciences

For a reference copy of the document with all sections, see nature.com/documents/nr-reporting-summary-flat.pdf

Life sciences study design

All studies must disclose on these points even when the disclosure is negative.

Sample size	More than 80 cells were counted and analyzed in each group in Figure 3A-F.<br>More than 31 cells were counted and analyzed in each group in Figure 3G-H.<br>More than 75 cells were counted and analyzed in each groups in Figure 4B-D.<br>More than 200 nuclei were counted and analyzed in each groups in Figure 5A-B.<br>More than 30 cells were counted and analyzed in each groups in Figure 6A-B.<br>More than 45 neurons were counted and analyzed in each groups in Figure 6C-D.<br>More than 52 neurons were counted and analyzed in each groups in Figure 6E
Data exclusions	No particular data were excluded.
Replication	Three biological replicates were carried out for experiments with statistical analysis.
Randomization	Not relevant to our study.
Blinding	All cellular images were captured under blind condition.

Reporting for specific materials, systems and methods

We require information from authors about some types of materials, experimental systems and methods used in many studies. Here, indicate whether each material, system or method listed is relevant to your study. If you are not sure if a list item applies to your research, read the appropriate section before selecting a response.

Materials & experimental systems

n/a	Involved in the study	
☐	☒	Antibodies
☐	☒	Eukaryotic cell lines
☒	☐	Palaeontology and archaeology
☒	☐	Animals and other organisms
☒	☐	Human research participants
☒	☐	Clinical data
☒	☐	Dual use research of concern

Methods

n/a	Involved in the study	
☒	☐	ChIP-seq
☒	☐	Flow cytometry
☒	☐	MRI-based neuroimaging

Antibodies

Antibodies used	anti-TDP-43 antibody (Abcam, ab104223), anti-Ran antibody (Abcam, ab155103), anti-importin-β antibody (Abcam, ab2811), anti-Lamin B1 antibody (Abcam, ab16048), anti-Nup153 antibody (Abcam, ab24700), and A11 antibody (ThermoFisher, AHB0052)
Validation	For all validations of antibodies could be found on the following websites:<br>https://www.abcam.com/tdp43-antibody-3h8-ab104223.html<br>https://www.abcam.com/ran-antibody-epr10791b-ab155103.html<br>https://www.abcam.com/kpnb1-antibody-3e9-ab2811.html<br>https://www.abcam.com/lamin-b1-antibody-nuclear-envelope-marker-ab16048.html<br>https://www.abcam.com/nup153-antibody-qe5-ab24700.html<br>https://www.thermofisher.com/antibody/product/Oligomer-A11-Antibody-Polyclonal/AHB0052

Eukaryotic cell lines

Policy information about cell lines

Cell line source(s)	SH-SY5Y, Cos-7
Authentication	SH-SY5Y was requested from Dr. Wen Zhi-Hong (Department of Marine Biotechnology and Resource, National Sun Yat-sen

Authentication	University, Taiwan) in 2017 and Cos-7 was requested from Dr. Pang-Hsien Tu (Institute of Biomedical Sciences, Academia Sinica, Taiwan) through collaboration in 2014.
Mycoplasma contamination	These cell lines were not tested by PCR; however, DAPI staining confirmed little or none Mycroplasma.
Commonly misidentified lines (See ICLAC register)	N/A
